# Contributing to health system resilience during pandemics via purchasing and supply strategies: an exploratory system dynamics approach

**DOI:** 10.1186/s12913-023-10487-7

**Published:** 2024-01-24

**Authors:** Paula Götz, Willem L. Auping, Saba Hinrichs-Krapels

**Affiliations:** Faculty of Technology, Policy and Management, Delft University of Technology, Jaffalaan 5, 2628 BX Delft, The Netherlands

**Keywords:** Critical medical supply chains, Health system preparedness, Health system responsiveness, Health system resilience, Personal protective equipment (PPE), System dynamics, Exploratory modelling and analysis

## Abstract

**Background:**

Health systems worldwide struggled to obtain sufficient personal protective equipment (PPE) and ventilators during the COVID-19 pandemic due to global supply chain disruptions. Our study’s aim was to create a proof-of-concept model that would simulate the effects of supply strategies under various scenarios, to ultimately help decision-makers decide on alternative supply strategies for future similar health system related crises.

**Methods:**

We developed a system dynamics model that linked a disease transmission model structure (susceptible, exposed, infectious, recovered (SEIR)) with a model for the availability of critical supplies in hospitals; thereby connecting care demand (patients’ critical care in hospitals), with care supply (available critical equipment and supplies). To inform the model structure, we used data on critical decisions and events taking place surrounding purchase, supply, and availability of PPE and ventilators during the first phase of the COVID-19 pandemic within the English national health system. We used exploratory modelling and analysis to assess the effects of uncertainties on different supply strategies in the English health system under different scenarios. Strategies analysed were: (i) purchasing from the world market or (ii) through direct tender, (iii) stockpiling, (iv) domestic production, (v) supporting innovative supply strategies, or (vi) loaning ventilators from the private sector.

**Results:**

We found through our exploratory analysis that a long-lasting shortage in PPE and ventilators is likely to be apparent in various scenarios. When considering the worst-case scenario, our proof-of-concept model shows that purchasing PPE and ventilators from the world market or through direct tender have the greatest influence on reducing supply shortages, compared to producing domestically or through supporting innovative supply strategies. However, these supply strategies are affected most by delays in their shipment time or set-up.

**Conclusion:**

We demonstrated that using a system dynamics and exploratory modelling approach can be helpful in identifying the purchasing and supply chain strategies that contribute to the preparedness and responsiveness of health systems during crises. Our results suggest that to improve health systems’ resilience during pandemics or similar resource-constrained situations, purchasing and supply chain decision-makers can develop crisis frameworks that propose a plan of action and consequently accelerate and improve procurement processes and other governance processes during health-related crises; implement diverse supplier frameworks; and (re)consider stockpiling. This proof-of-concept model demonstrates the importance of including critical supply chain strategies as part of the preparedness and response activities to contribute to health system resilience.

**Supplementary Information:**

The online version contains supplementary material available at 10.1186/s12913-023-10487-7.

## Introduction

The purpose of a health system is to “improve, maintain or restore health” [1], but this ability can be severely impaired during disruptions such as a pandemic. During the COVID-19 pandemic, health systems worldwide struggled to deliver basic services [2, 3]. This prompted policy and scholarly work to focus on resilience of health systems [4–6]. Resilience is often described using the following characteristics: preparedness, response, recovery, and growth [7, 8]. Given that critical medical supplies, such as drugs, devices and equipment, are considered one of the essential building blocks of any health system [9], it is just as important for their associated supply chains strategies to enable the health system to be prepared and responsive in an emergency. However, the COVID-19 pandemic showed that health systems struggled to organize sufficient critical medical supplies such as personal protective equipment (PPE) and ventilators [10, 11].

Today’s critical medical supply chains tend to focus on creating efficiencies through cost minimization, the reduction of inventory, and the maximization of utilization across the entire supply chain, aiming for so-called ‘lean’ strategies [2, 3, 12]. A potential negative consequence of creating efficiencies, however, is that the supply chains’ abilities to respond to unexpected events decreases: maintaining a low inventory, for example, decreases preparedness as it does not allow for sudden increases in demand [13, 14]. Furthermore, PPE and ventilator supply chains operate in a globalized and interconnected world [15], meaning that global disruptions, such as transportation network disruptions or export bans, can significantly affect critical medical supply chains. Consequently, lean critical medical supply chains that are prone to uncertainties combined with global disruptions are likely to be significantly affected by pandemics [3, 12].

During the onset of the COVID-19 pandemic, many health systems in the Global North used two types of strategies to purchase and supply PPE and ventilators: preparedness strategies and responsiveness strategies. Preparedness strategies focus on the time frame before a shock hits the health system and focus on long-term solutions [2, 7, 8]. The most popular supply strategy applied in preparedness is stockpiling, as it helps to absorb any demand shocks in the beginning of a crisis [16–18]. On the other hand, responsiveness strategies focus on the time window when the crisis first occurs [2, 7, 8], which is when the health system’s performance is heavily affected. In the context of the Global North, decision-makers in health systems often decided to “ramp up” domestic production [19, 20], support innovative supply strategies [21–23], or to loan products from other sectors such as the private health sector [24] as a response. Additionally, health systems could purchase products from the world market in competition with other health systems, or using accelerated processes without competition through direct tender [25]. Many health systems applied these supply strategies simultaneously.

However, to our knowledge, there are few academic studies that demonstrate the effects of these different purchasing and supply strategies for critical medical supplies in preparing and responding to a pandemic situation for a specific region. Existing literature has focussed on mitigation strategies for managing whole supply chain disruptions during COVID-19 [26] or on categorising supply chain research management more generally [12, 27]. As pointed out by Sigala et al. (2022), few studies take a systemic approach to risk management in the supply chain literature [28]. Their study provided a more systemic view on the effects of PPE supply chain disruptions during the first wave of COVID-19 across regions and countries. In this study, we specifically assess the health system effects of different national purchasing and supply strategies for acquiring PPE and ventilators during the beginning of pandemics, using an exploratory [29, 30] System Dynamics (SD) approach [31].

We use the term critical medical equipment or critical medical supplies interchangeably to refer to medical supplies, such as PPE and ventilators, during health-related crises. A health-related crisis is a “disruption that physically affects a (health) system as a whole and threatens its basic assumptions, its subjective sense of self, its existential core, … and it triggers a public policy chance” [32].

In consecutive sections, we focus on the methods used in this research, the results of this work, followed by a discussion focusing on the uncertainties affecting different supply strategies, and conclusions from our exploratory study.

## Methods

To simulate and analyse the success of different supply strategies under various scenarios, we used SD in combination with Exploratory Modelling and Analysis [29, 33]. First, we used a SD model to model the spread SARS-CoV-2, using data from the English national health service (NHS), the resulting demand for PPE and ventilators, as well as the supply strategies. To explore the impact of uncertainties on the shortage of PPE and ventilators, we applied Exploratory Modelling and Analysis to the SD model. We introduce the research methods and explain our motivation to use them below.

### System dynamics to model the spread of COVID-19 and supply strategies

SD is a deterministic, differential equation-based simulation modelling method with a strong visual language. It is extremely suitable for understanding problems within complex systems, such as the organization of critical medical supplies, as it centres around the idea that a problem is present due to the interactions, feedback loops, and delays among different components within a system [34]. These characteristics cause the non-linear behaviour of systems, which can be simulated with SD models over a certain time horizon. Also, SD offers the chance to find bottlenecks that cause problems within systems [35]. Problems that are suitable to be modelled using SD include questions related to accumulation, an increase or decrease of a quantity over time, delays (meaning that the effect of one variable on another is delayed), and feedback loops (meaning “when the effect of a causal impact comes back to influence the original cause of that effect” [36]). That is the case for the provision of PPE and ventilators during a health-related crisis like COVID-19, as it is characterised by accumulation phenomena such as the stockpiling of PPE and ventilators, delays such as those caused by procurement processes, or the time between the production and delivery of PPE, as well as feedback phenomena such as the interplay between the infectious and the susceptible population.

In previous studies, SD has continuously proven to be a useful method to address the complexity of problems within health systems [35, 37–40], for instance, focusing on health service delivery or health care capacities and their fluctuations. Within existing literature focussing on supply chains for health systems, only a handful of studies used SD for problems relating to critical supply availability [41–43]. SD modelling in supply chain management often focuses on decisions regarding inventory levels, demand amplification, supply chain design, supply chain management, sustainability, as well as stockouts of medicine [44–46], but there has been less focus on using SD to combine procurement processes, stockpiling, distribution, and forecasting of PPE and ventilator usage, as well as the modelling of the supplier side of PPE and ventilators.

The base for our SD model was a S(usceptible) E(xposed) I(nfected) R(ecovered) transmission model to present the spread of COVID-19 and the resulting demand for critical medical supplies, in this case PPE and ventilators. The SEIR model, demonstrated to be applicable for modelling disease spread for different scenarios [47], was used as a deterministic transmission model. In the SEIR structure, we differentiated between different country groups (i.e., England, high-income countries, middle-income countries, low-income countries) and different age cohorts to represent the age-dependent severeness of COVID-19. The SEIR model was extended with stocks to represent the hospital population in intensive care and general wards. We assume that the demand for PPE and ventilators is derived from the demand for general hospital/intensive care units.

Next to the SEIR sub-system, several other sub-systems are included in the SD model. One sub-system focuses on the demand for PPE and ventilators at each simulated time step, and a forecasted demand for further time steps. That information is fed into the sub-system focusing on the decision-makers’ ordering process of placing orders with different supply strategies. An overview of the differences in the supply strategies used in our model is provided in the Table 1.

**Table 1:** Relevant characteristics of six supply strategies: direct tender, purchasing from the world market, stockpiling, domestic production, innovation, and loaning; as used in our model

	**Direct Tender**	**Purchasing from the World Market**	**Stockpiling**	**Domestic Production**	**Innovative Supply Strategy**	**Loaning**
Definition	Decision-makers can place orders with suppliers using accelerated processes and without competition [25]	Decision-makers can order products from suppliers from the world market. The English health system is in competition with other health systems [25]	Decisionmakers can implement and operate stockpiles with critical medical supplies. In England, Public Health England created a PPE stockpile designed for an influenza [25]	Decision-makers reached out to companies to start the production of critical medical supplies [25]	Individuals or companies produce critical medical supplies in small batches, such as highlighted in the article by [48]	Decision-makers can loan critical medical supplies from other sectors if they fit in the context. In England limited to ventilators from private medical sector [24]
Production Capacity	Assumption that production capacity equals 5% of production capacity of suppliers reached through world market. Production capacity can be increased with increasing demand [49]	Highest production capacity compared to other supply strategies. Production capacity is based on sources where possible. Production capacity can be increased with increasing demand [49]	Limited to the inventory stored in the stockpile. Initial values were derived from [25]	Assumption that production capacity equals 60% of suppliers reached through direct tender for PPE. Concerning ventilators,”ventilator challenge” in England is used as a base [24]. The initial production capacity is 0 and increases over time	Production capacity is small per project and increases over time as more products are approved. Assumptions based on information about single projects [48, 50] or on information on the”ventilator challenge” [24]	Limited to the availability of critical medical supplies in other sectors. In England, 1200 ventilators were loaned private medical sector [24]
Shipment Time	Shipment time is assumed to be 1.5 months, varies between 1 and 3 months [51]	Usual shipment time around 3 weeks. During height of the COVID-19 pandemic, shipment times of up to 9 months [51]	Shipment within 5 days [52]	Shipment within 5 days Based on information about PPE delivery times by [52]	Shipment within 5 days	Shipment within 5 days
Vulnerability	Share of unusable critical medical supplies purchased [25]	Export restrictions Delayed shipment times [25]	unknown	unknown	unknown	unknown
Input of Decision-Makers	Setup time Order buffer	Setup time Order Buffer	Equipment of stockpile Operations of stockpile	Setup time Order Buffer	Setup time Government support Order buffer	Setup time

Another sub-system models the considered supply strategies focusing on their supply chains, including shipping times and supply chain performances. To measure the success of supply strategies, the normalized shortage or coverage per day and in total over time is measured. To account for different kinds of PPE, we differentiated between five different types (i.e., gloves, simple face masks, respirators, gowns, and eye protection). Differences are present in the production capacities as well as other supply chain factors.

### SD Model: extended SEIR model to add supply strategies

**Fig. 1:**
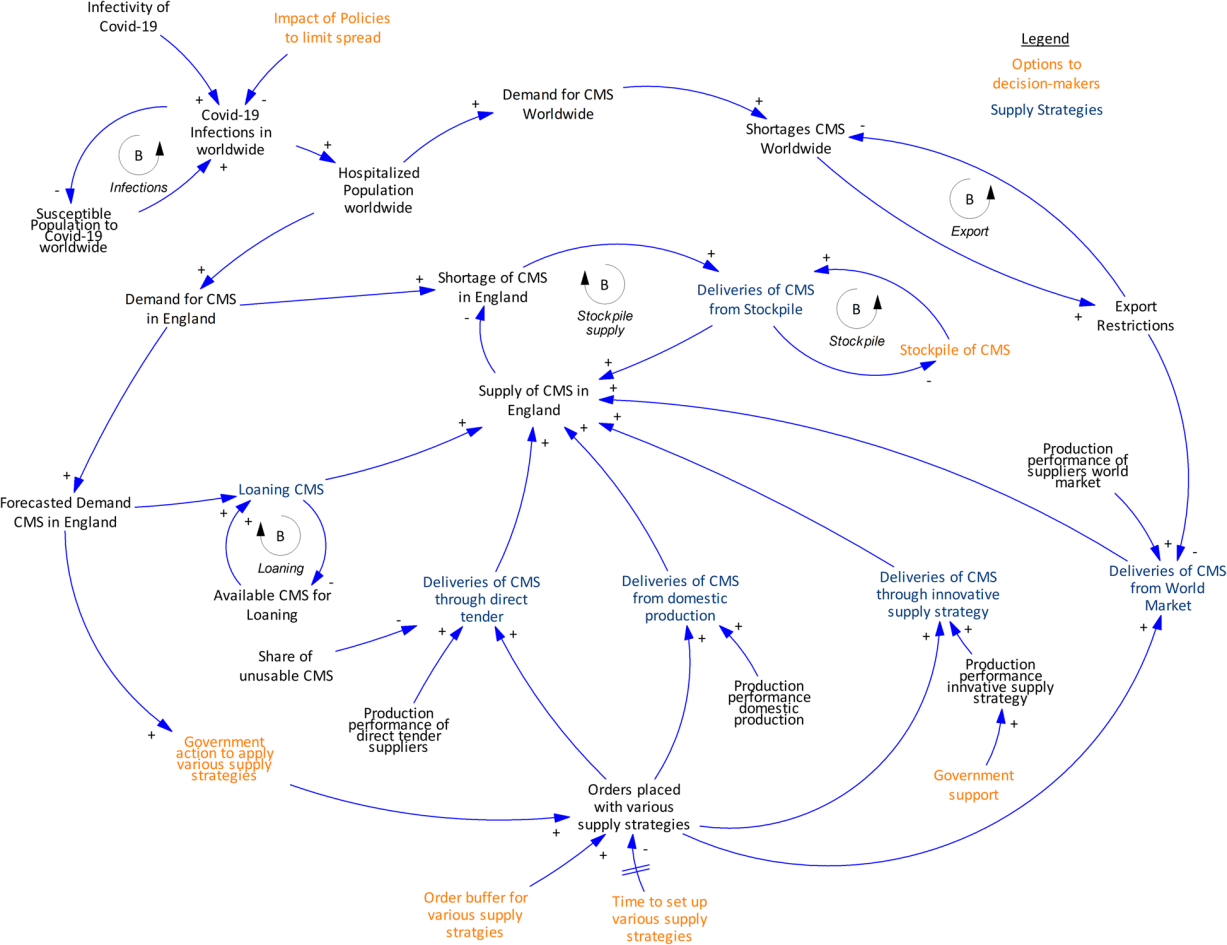
Causal Loop Diagram showing high-level conceptual overview of supply chain model, showing only the most important relationships in the system. Loops are highlighted with circular arrows. Loops can either balance or reinforce the behaviour of the system. (CMS: critical medical supplies)

We constructed an exploratory SD model [30] based on the collected knowledge from the case study (the English NHS). The most important relationships in the system are illustrated in a causal loop diagram (Fig. 1) [53]. A causal loop diagram captures the most critical components of the model and helps to grasp the relationships between the variables within a system. Arrows with ( +) indicate a positive relationship (when one variable increases, so does the other) and those with (-) indicate an opposite relationship (when one variable increases, the other decreases, and vice versa).

Based on the causal loop diagram, one could follow the following train of thought to explain the behaviour of the system as the dynamic hypothesis. As COVID-19 spreads throughout the population, the pressure on hospitals and the demand for PPE and ventilators increases. Once the demand for PPE and ventilators exceeds the existing supply in the health system, a shortage appears for critical medical supplies. This is a call for decision makers to order critical medical supplies or set up various supply strategies (Table 1): (i) purchase through direct tender, (ii) purchase critical medical supplies from the world market*,* (iii) stockpile, (iv) start domestic production, (v) support innovative supply strategies, or (vi) loan ventilators from the private sector (shown in blue text, Fig. 1). Decision-makers can decide about the combination of supply strategies to apply, how orders should be placed, and they can implement different actions to reduce the set-up time of supply strategy (shown in orange text, Fig. 1). Such actions can include creating and implementing plans about how to act in times of crisis. How many critical medical supplies end up reaching the country (in this case, England) depends on factors affecting the resilience of a supply strategy, such as export restrictions or the risk of unusable products being delivered. As more critical medical supplies are provided, the shortage of critical medical supplies decreases in the country.

We performed various tests to check whether the model was fit for purpose [34, 54, 55]. Specific tests that we performed, based on Forrester and Senge [54], were on structure verification, parameter verification, extreme conditions, boundary adequacy, dimensional consistency, behaviour reproduction, behaviour anomaly, behaviour sensitivity, and policy sensitivity. We found that our results generally hold true, apart from countries where due to large income disparities the GDP per capita is not a good measure of the development level or the whole population.

### Exploratory modelling and analysis to find successful policies

According to Bankes [29], exploratory modelling is a method to explore the impacts of different uncertainties and hypotheses regarding the model structure. For such an exploration, computational experiments (i.e., large amounts of model runs) that cover the range of values of uncertainties in the model are used. Exploratory modelling was thus deemed suitable to apply to the modelling of critical supply chains in health systems, since numerous uncertainties affect the functioning of health systems and its supply of critical medical supplies during pandemics such as COVID-19 [56, 57]. Such uncertainties can include, for instance, export restrictions or production capacities. We therefore used Exploratory Modelling and Analysis (EMA), as it allows us to analyse the influence of parameters on model outcomes under different assumptions.

To identify the uncertainties affecting the model’s outcome significantly, as well as the characteristics of successful policies, we applied Many Objective Robust Decision-Making (MORDM) [58]. In our case, we defined a successful policy as one that minimizes the shortage of PPE and ventilators. To apply MORDM, we used the EMA Workbench [59], an open-source library for Python, to conduct the series of computational experiments. The MORDM process consists of four steps: problem formulation, alternative generation, uncertainty analysis, and scenario discovery [60].

Before the MORDM process is applied using the EMA workbench, we conducted an open exploration to explore how the different policies behave over the range of uncertainties. A policy is a combination of different supply strategies and set-up times for each supply strategy. The results of the open exploration are visualised using graphs with the dynamics of all model runs. For instance, feature scoring was used to identify the differences in impact uncertainties have on the outcome [59].

The first step of the MORDM process is the problem formulation. It contains the definition of uncertainties, levers (decision variables), and the model outcomes, which can also be described as measures of performance [58]. For this step, it is vital to identify the range of values that both uncertainties (Table 2) and decision levers (Table 3) can take. A detailed overview of all decision levers, outcomes, and uncertainties can be found in the digital appendix (https://github.com/paulagoetz/resilientsupplystrategiespandemicsSD).

**Table 2:** Overview of uncertainties and associated assumptions/sources used in model

Category	Uncertainty	Source
Supply Chain	Base raw material & production capacity for PPE and ventilators. Increase in production capacity over time for PPE and ventilators	Assumptions made based on sources where possible. For instance, information by [23, 48, 49, 61]
Supply Chain Disruption	Shipment times; Export Restrictions; Delays in the production & procurement of products	Values obtained from information by [25, 51]
Crisis	Infectivity	Values obtained from information by [62, 63]

**Table 3:** Overview of decision levers and associated assumptions/sources used in model

Decision levers	Definition
Switches for supply strategies	These variables determine whether a supply strategy is applied or not. Stockpiles are assumed to be always in place
Order buffers for supply strategies	Order buffers are applicable to supply strategies, excluding stockpiling PPE or ventilators. Order buffers determine to what extent a supply strategy is prioritized. Higher-order buffers trigger higher sizes in orders
Setup times for supply strategies	Setup times determine how quickly orders can be placed with a specific supply strategy. Setup times are not considered for stockpiles. More effective procurement and supplier frameworks can reduce the setup times for supply strategies
The effectiveness of innovative supply strategies	Decision levers in this category affects the impact of the supply strategy innovation. Levers in this category include, for instance, government support
The composition and operation of stockpile	Decision levers in this category focus on the logistics of the stockpile and its equipment
Operational levers	In the developed system, it is assumed that products are stored centrally before being delivered. These variables focus on the effectiveness of these processes
Levers affecting forecast and order point	Levers in this category include the forecast horizon and the threshold value to order PPE

During the alternative generation, a set of candidate policies is generated based on a specific state of the world, which is defined by the value of uncertain variables and is referred to as a scenario. The scenario of interest in our research is the worst-case scenario identified in the open exploration. We found the candidate policies using multi-objective optimization by identifying the best policies given the worst-case scenario. The optimization uses Latin hypercube sampling [64], as suggested by Bryant and Lempert, 2010 [60] since it provides an efficient sample of a model’s behaviour.

Afterwards, an uncertainty analysis is conducted by running experiments over the identified candidate policies and different scenarios [58]. The range of values for the uncertainties needs to be reasonable but does not always have to be possible [58]. We used the uncertainty analysis results as an input for the scenario discovery and to compare the candidate policies in terms of robustness.

Lastly, scenario discovery is conducted to identify combinations of uncertainties that have the most significant impact on the outcomes of the model. In that way, we can discover by what circumstances our results are affected the most. Scenario discovery uses clustering analyses to find combinations of uncertainties that best predict an outcome of interest [60]. The outcome of interest is low coverage of PPE and ventilators for the case at hand. For the scenario discovery, the EMA workbench deploys the PRIM algorithm [65]. After the application of the PRIM algorithm, the user can choose a scenario based on its coverage and density value. A high coverage indicates that the selected scenario can explain a high share of outcomes of interest, a high value in density indicates the certainty in the prediction [58]. During the scenario inspection, we can identify the most relevant uncertainties affecting the outcome of interest, and their range of values [58]. Additionally, we can visualize the results using dimensional stacking, which identifies the combination of uncertainties that have the most impact on the outcome of interest.

### Experimental design

In all our experiments, we used Vensim DSS version 9.2.4 in combination with EMA workbench release 2.3. We simulated the model over a time horizon of 210 days (in the beginning of the pandemic), with a time step of 0.0078125 day, and Euler integration method. We performed three different sets of experiments with our model. First, we did an open exploration in which the four previously designed policies were tested over 800 scenarios resulting in 3200 experiments considering the uncertainties. As the policies did not show a significant improvement, a directed search for PPE and ventilators to generate a set of candidate policies that perform best considering the worst-case scenario identified in the open exploration was conducted. The directed search was run over 4000 “number of evaluations” for ventilators and 5000 for gloves & simple masks aiming to maximize the total coverage. To track the convergence of the optimization process in the directed search, we tracked the ϵ-progress using an ϵ-value of 0.05. The directed search resulted in six candidate policies.

Second, we performed an uncertainty analysis using the candidate policies identified in the directed search and the defined uncertain variables. For these runs, we reduced the amount of uncertainties considered, as PRIM’s scenario discovery can only consider up to around 30 uncertainties. Uncertainties associated with a specific supply strategy not relevant to the candidate policies and uncertainties affecting the spread of the virus were eliminated. Feature scoring of the open exploration showed that uncertainties affecting the spread of COVID-19 had a significantly higher impact and outshone uncertainties relevant to supply strategies. The uncertainty analysis considered 1000 different scenarios. Hence, for the case of ventilators, 1000 experiments and in the case of simple masks & gloves, 6000 experiments were run.

Third, we did scenario discovery using PRIM to identify the variables that have the most impact on the changes in performance, considering the case of ventilators. To do so, one must define performance measure thresholds regarding the outcomes of a model. The performance threshold was chosen to focus on values lower than 35% of worst cases regarding the total coverage regarding ventilators, simple masks, and gloves. PRIM returned the uncertainties most likely to contribute to the outcome and their range of values.

## Results

We include three experiments in the results of our simulations: (a) open exploration, where we explore the behaviour of the system; (b) testing the results of alternative policies; and (c) testing these policies under different future uncertain scenarios.

### Open exploration: exploring the behaviour of the system

**Fig. 2:**
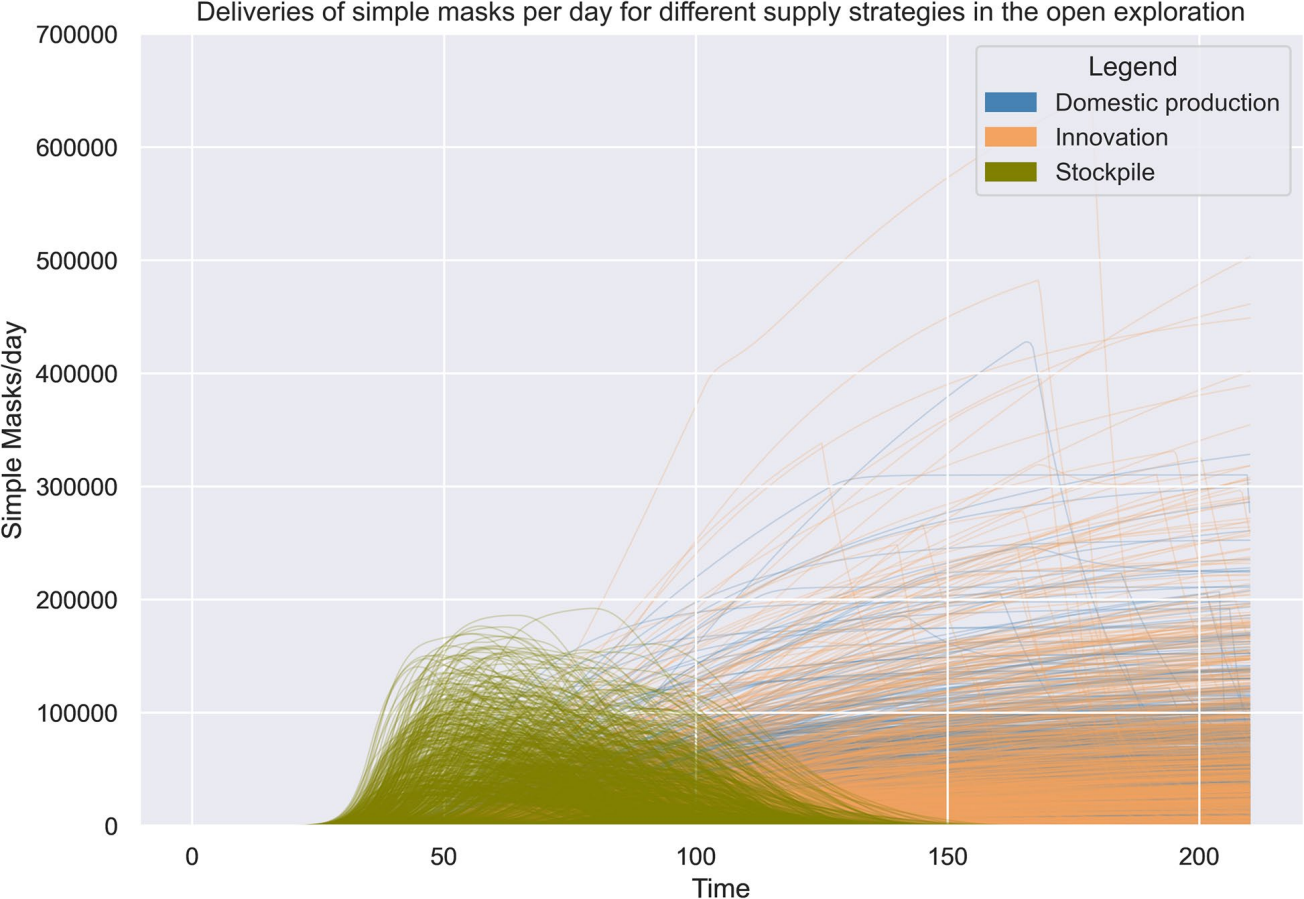
Comparison of deliveries between different supply strategies over time. Each line represents one experiment. **a** represents the supply strategy domestic production, **b** represents the supply strategy stockpiling, **c** represents the supply strategy innovation. The y-axis on each figure represents the delivered products per day

**Fig. 3:**
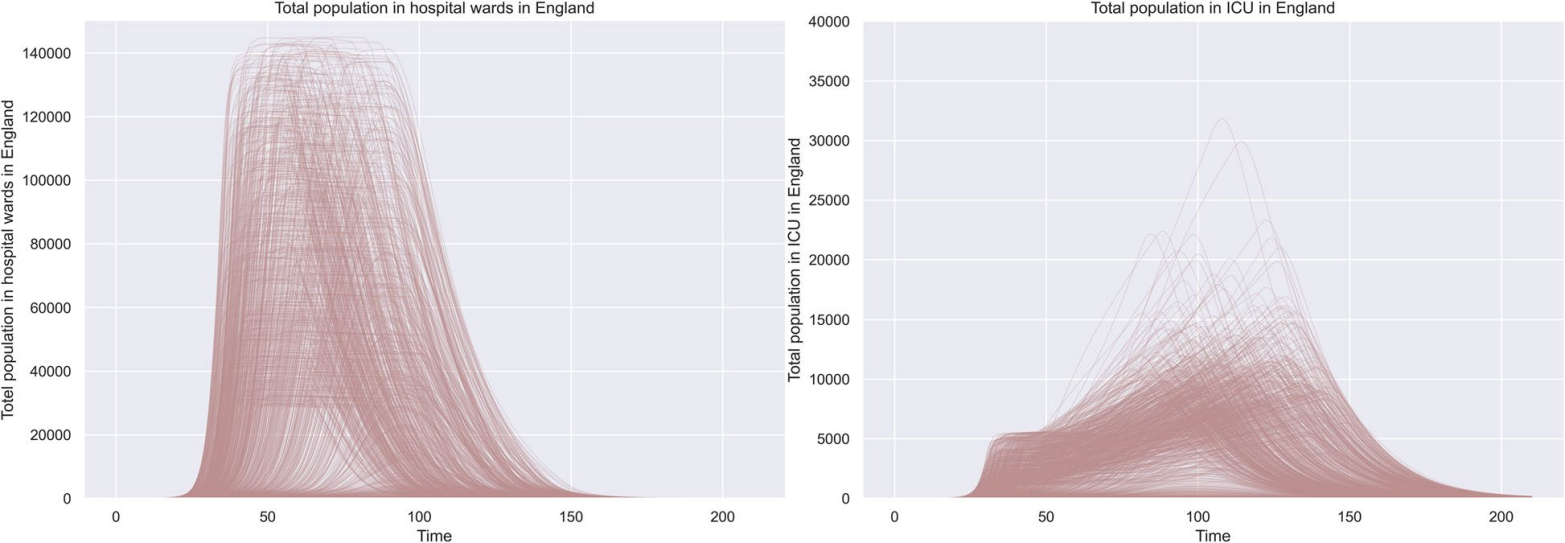
Simulated behaviour for hospitalized population (**a**) and the population in ICU (**b**)

The results of the open exploration indicate that a long-lasting shortage in PPE and ventilators is likely to be apparent in various scenarios (Fig. 2). Looking at the impact of the different supply strategies, we could observe that: (i) stockpiling helps to absorb the first demand shocks of PPE, as the graphs shows that the deliveries start significantly earlier compared to other supply strategies. (ii) Supply strategies purchasing PPE and ventilators from the world market and purchasing PPE and ventilators through direct tender can contribute the most to decreasing the shortage of ventilators and PPE. The highest volume of deliveries is possible through the supply strategy purchasing products from the world market. (iii) “Ramping up” domestic production and focusing on innovation as supply strategies can contribute significantly to the availability of PPE and ventilators during the beginning of COVID-19, but at a lower level than other supply strategies and at a later point in time. The graphs indicate that the spread in deliveries is higher. (iv) Loaning ventilators from other sectors can help to absorb arising demands in the beginning of COVID-19 due to its low delivery times. The capacity of the supply strategy is limited. An overview of the potential impact of the supply strategies domestic production, innovation, and stockpiling can be seen in Fig. 2. For comparison, the number of hospitalisations over time is shown in Fig. 3.

### Characteristics of candidate policies

We define a “candidate policy” as the combination of different decision variables. The alternative generation resulted in candidate policies that exhibited differences in the combination of applied supply strategies and set-up times. The set-up time refers to the time it takes before orders have reached the supply strategy, which requires a fast reaction by decision makers. As expected, the results show that the candidate policies exhibit very low set-up times for the applied supply strategies. Furthermore, we found out that all candidate policies applied stockpiling as a supply strategy and for most candidate policies it was suggested to increase the inventory level to a higher level than originally recommended.

**Fig. 4:**
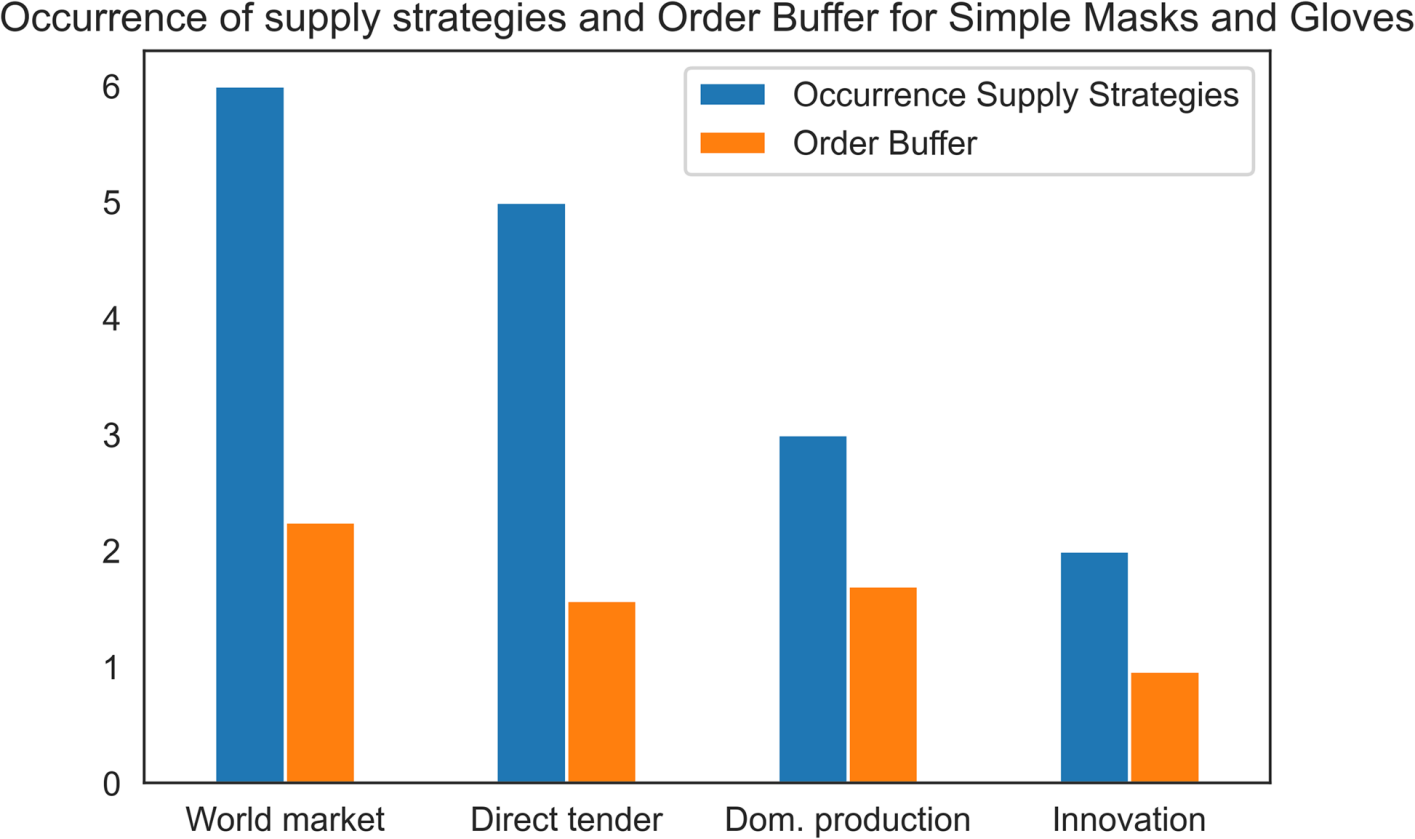
Occurrence of supply strategies in the candidate policies for simple masks and gloves. Six candidate policies were identified overall, and all candidate policies applied the supply strategy purchasing from the world market. The order buffer indicates how much decision-makers prioritize/ overorder products

We found out that the supply strategies purchasing products from the world market and purchasing products through direct tender were applied in almost all candidate policies, as shown in Fig. 4. In contrast, the supply strategies focusing on supporting innovations and domestic production were applied fewer times within the candidate policies for the considered PPE, simple masks and gloves. That means considering the worst-case scenario it is less attractive to the English health system to produce PPE domestically or through innovative ideas compared to purchasing products from the world market or purchasing products through direct tender.

### Scenario discovery: impactful uncertainties

**Fig. 5:**
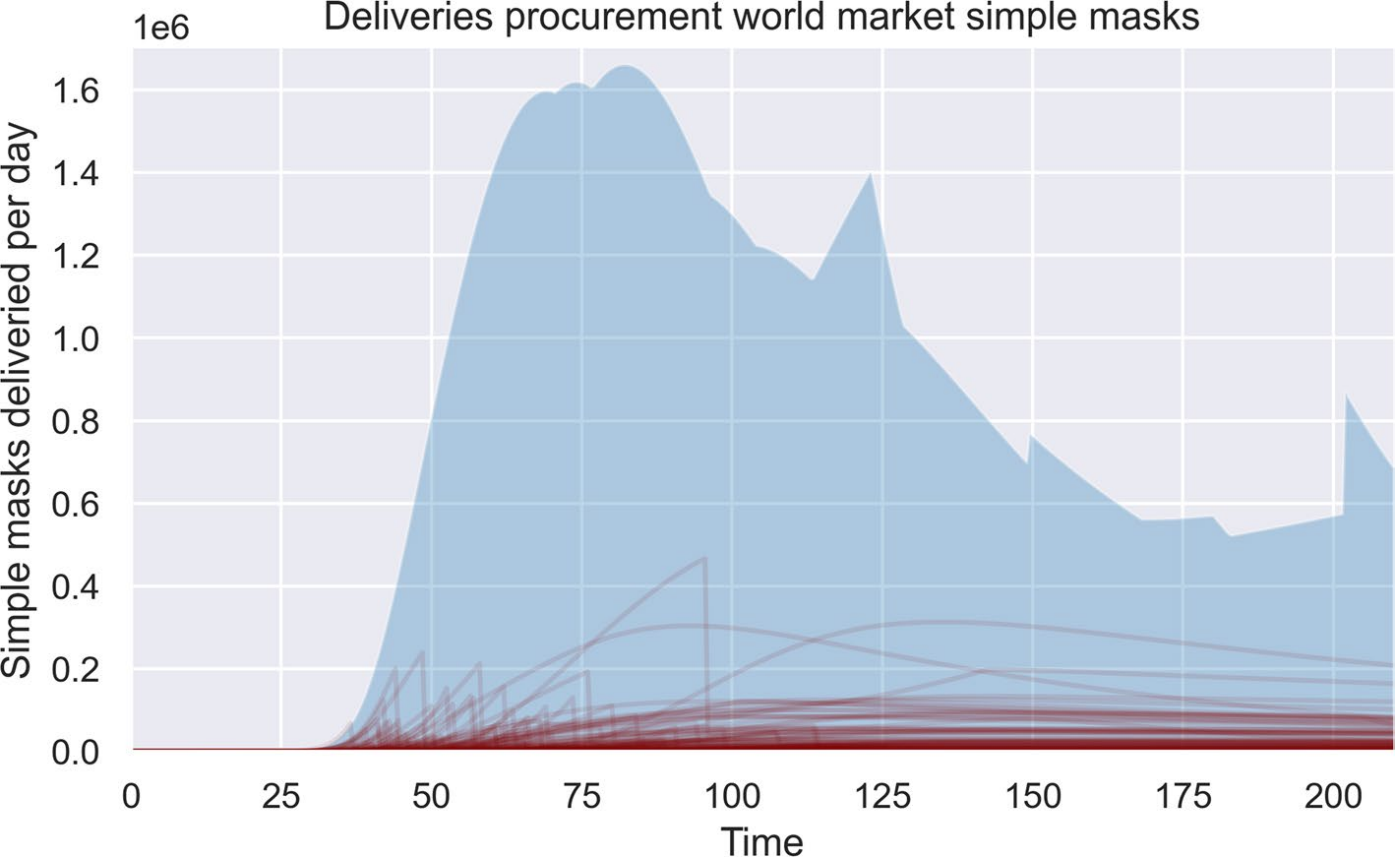
Impact of export restrictions. Experiments with extreme export restrictions are highlighted in red. The range of possible deliveries per day for all cases is shaded in light blue

**Fig. 6:**
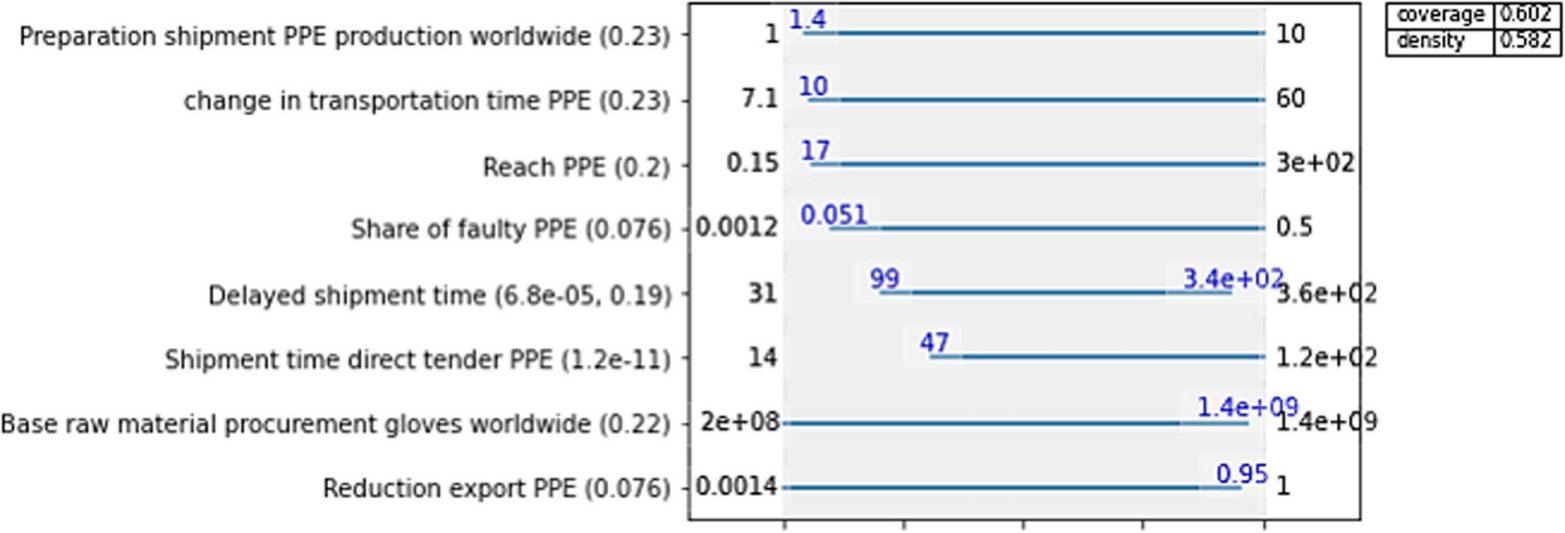
Uncertainties that the define the scenario of interest for gloves. The plot indicates what range of values potentially result in a low availability of gloves and lists the most influential uncertainties on the left. The value in parenthesis next to the uncertainties is the qp-value. A lower qp-value indicates how influential an uncertainty is. Hence, the most significant uncertainties in that case are: The share of unusable PPE delivered by suppliers reached through direct tender, delayed shipments from suppliers reached through the world market, delayed shipments from suppliers reached through direct tender, and any export restrictions concerning the suppliers reached through the world market

Using the scenario discovery, we analysed which uncertainties affect the success of candidate policies the most. Since this work focuses on actions of health systems during crises, we are specifically interested in which scenarios the candidate policies perform the worst. We found out that especially uncertainties surrounding the supply strategies purchasing products through direct tender or purchasing products from the world market impact the low availability of simple masks, gloves, and ventilators (only direct tender for ventilators). Overall, the most influential uncertainties were high shipment times, delays in the production of the different supply strategies, and export restrictions (Fig. 5). Figure 6 specifically focuses on the case of gloves and indicates what uncertainties have the most influence on a low availability of gloves.

## Discussion

In our discussion, we focus on the factors contributing to the impact of candidate policies and the role of stockpiling. We also discuss the results of the scenario discovery and how the most impactful uncertainties could be mitigated. Additionally, we explore possible additional supply strategies that could be tested in the future based on literature.

### Set-up times as an enabler for the success of responsive supply strategies

As expected, we found out that short set-up times for supply strategies are required for candidate policies. This means that decision makers need to take actions in preparedness for pandemics and other crises to lower set-up times. Decision-makers can apply crisis frameworks to enable short set-up times. Crisis frameworks accelerate procurement and governance processes, by providing plans of action concerning the implementation of different responsive supply strategies [21, 66–68]. This can include clear coordination frameworks, accelerations by including direct awarding of contracts, or setting up collaboration frameworks. To specifically reduce the set-up time of the supply strategy of domestic production, raw materials can be stored to start domestic production quickly.

### The role of stockpiling

Our results suggest that stockpiles should be equipped at a higher level. However, raising the inventory level raises the question about how to best balance costs or lean approaches, compared to a higher level of preparedness [69, 70]. Costs are often used as an argument against implementing a high level of stockpiles, but Dow et al. 2018 [17] point out that in case of COVID-19 overall costs for PPE would have been lower if a stockpile had been in place versus if all PPE were purchased during an emergency. Hence, it is necessary for decision makers to investigate further how to best balance costs and the level of preparedness.

Additionally, the maintenance of stockpiles and composition of the “right” products can be a challenging task for decision-makers in health systems [71–73]. Expired products need to be removed and the stockpile needs to be updated when necessary [74]. As expected, we found out that candidate policies did not allow for delays in the operationalisation of the stockpile, meaning that operations within warehouses (e.g. preparing PPE for delivery, packaging and sorting PPE) must run smoothly, and supplies must be delivered promptly. Overall, decision makers must realize that a stockpile’s operation and composition heavily affect the success of stockpiling as a preparedness strategy.

### Mitigating uncertainties

During scenario discovery, we found that especially uncertainties associated with the supply strategies purchasing critical medical supplies through direct tender and purchasing critical medical supplies from the world market are impactful due to their high production capacities. Hence, it is crucial for decision makers to mitigate uncertainties surrounding these supply strategies. Delayed shipment times and varying production performances can negatively influence both supply strategies’ success. To mitigate these uncertainties, health systems can diversify their supplier framework, as it can help to ensure deliveries during times of crisis, echoing recommendations from previous studies, such as Hanfield et al. 2010 [26] and Sodhi et al. 2021 [75]. The production of PPE and ventilators is focused on a few areas worldwide. By purchasing products from various suppliers, health systems may be able to ensure shipments and reduce delayed shipments. A diverse framework can help to increase the number of reliable suppliers; and it can help to mitigate the effect of export restrictions if critical medical supplies are purchased from the world market.

However, JIang et al. 2022 [14] raise the valid question of how much a diverse supplier framework can help if the entire network is affected by transport restrictions causing delayed shipment times. Hence, the advantage may only be temporal. Currently, it is assumed in our model that decision-makers place orders with different supply strategies without considering the supply strategies’ performance. Implementing an information system could make decision-makers more flexible with placing orders with the most adequate supply strategy/suppliers. Also, choosing adequate suppliers can reduce the share of unusable critical medical supplies.

Our developed model showed that the uncertainties present during the COVID-19 pandemic and numerous other scenarios can cause serious impacts on the availability of PPE and ventilators in hospitals. Furthermore, it stands out that decision-makers often cannot influence the impact of uncertainties directly, but they can apply measures to reduce their effect.

### Additional supply strategies to consider further

Since the results of our research indicate that shortages are likely to be present under numerous scenarios, it is necessary for decision-makers to keep in mind that the considered supply strategies may not be successful and additional supply strategies need to be considered. Amongst others, decision-makers could start to loan ventilators from other sectors and create a database with available ventilators in the country. Additionally, decision-makers can implement open collaboration frameworks to innovate PPE products, invest in research regarding new products, or increase the reusability of PPE. Another strategy that could be tested is the sharing PPE across countries or developing a global stockpile of medical equipment as proposed by Dey et al. 2020 [76].

### Further research and limitations

Concerning the developed SD model, it is important to further improve the decision framework and forecasting method. The decision framework currently does not consider any planned deliveries and the exactness of the forecast needs to be improved due to its simplicity. Furthermore, additional factors, such as export restrictions for the supply strategy direct tender, are currently not considered.

Concerning the Exploratory and Modelling Analysis, a major limitation is that the selection of candidate policies is solely based on the worst-case scenario, which can result in policy interventions that could be considered ‘overkill’, or too drastic for most likely scenarios. Hence, for future research, the alternative generation should be based on a set of different scenarios to receive candidate policies. Additionally, due to limited resources, not all PPE products were considered, and, due to technical limitations, not all uncertainties were handled by the PRIM algorithm during the scenario discovery. Hence, these two factors need to be considered for future work. Additionally, we made the decision to consider the worst-case scenario for the MORDM process and the policies were not further assessed over a range of scenarios. For future work this should be considered as current supply strategies might over perform in many possible scenarios. Finally, it would be interesting to test the validity of this model for other types of disasters in future research.

Regarding the system understanding, the following further research is suggested. In this research, we understand the English health system as an independent actor in competition with other countries. The impact of sharing equipment across health systems worldwide should be investigated in a separate model. Furthermore, this model does not consider costs as one of its outcomes. For future work, costs should be considered as an additional outcome and the policies should be optimized considering their success in decreasing the shortage of medical supplies and minimizing costs.

For further research we suggest including the healthcare workforce as a resource of need next to considering solely critical medical equipment. In that case the model structure would have to be extended to include the need for workers as well as the effect of the COVID-19 pandemic on the workforce. Lastly, we suggest going into more depth regarding the modelling of the specific supply strategies, as they are currently modelled at a very high level.

We stress that this study was exploratory in nature, showing a proof-of-concept of using SD and EMA to demonstrate the effects of different supply chain strategies for improving availability of critical medical supplies for health systems within crisis situations. Future models, based on this exploratory version, could be developed with appropriate data sources and validation of our initial model, to enable to development of future interfaces that can then support decision-making.

## Conclusions

In this study, we developed a proof-of-concept simulation model that demonstrates how health systems can acquire critical medical equipment during pandemics, using the case of the supply of PPE and ventilators during COVID-19. Our model connects a disease transmission model with the resulting demand and supply of PPE and ventilators within hospitals of a health system and simulates the effects of different supply strategies for a large number of scenarios.

Based on our preliminary results in our exploratory model, we found out that the time to set-up supply strategies and uncertainties surrounding the selected supply strategies affect the shortage of PPE and ventilators. The types of strategies that show potential for improving the preparedness for pandemics to ensure the availability of PPE and ventilators during pandemics include to (i) develop crisis frameworks that propose a plan of action and consequently accelerate and improve procurement processes and other governance processes during health-related crises, (ii) implement a diverse supplier framework, that sources products from different parts of the world, so that health systems are less affected by transportation problems, (iii) implement an information system that enables the information exchange between suppliers and health systems, so that decision-makers are able to place orders with the most adequate supplier, and (iv) store raw material for the domestic production of critical medical supplies, so that the set-up time of domestic production is lower. Although these results are based on a proof-of-concept simulation, our main intention is to demonstrate the importance of including critical supply aspects of health service provision within the broader preparedness and response strategies for health systems resilience.

### Supplementary Information

**Additional file 1.**

**Additional file 2.**

## Data Availability

The full assumptions in the entire model are in the Additional files A and B. The entire model is presented in a public online repository available at: https://github.com/paulagoetz/resilientsupplystrategiespandemicsSD.

## Abbreviations

COVID-19: Coronavirus disease, an infectious disease caused by the SARS-CoV-2 virus
SD: System Dynamics
SEIR: Susceptible, Exposed, Infected, Recovered
EMA: Exploratory Modelling and Analysis
