# Supplementary material for: Contributing to health system resilience during pandemics via purchasing and supply strategies: an exploratory system dynamics approach
Source: BMC Health Serv Res. 2024 Jan 24;24:130. doi: 10.1186/s12913-023-10487-7 (PMC10807148; doi:10.1186/s12913-023-10487-7)
Supplement: Supplementary file 1 — Additional file 1. [file 12913_2023_10487_MOESM1_ESM.pdf]

A

Model Overview

This Appendix presents the created Vensim model and its subsystems. In Figure A.1, you can find how different parts of the model presented in the following pages are connected to each other.

How many people are infected in the SEIR model, presented in Figure A.2, depends on the public behaviour regarding contact restrictions (A.3, A.4, A.5), and the susceptible population. How many people can be treated in hospitals in the SEIR model, depends the hospital capacities (A.6). On the other hand, the SEIR model determines the demand for PPE and ventilators (A.7, A.8). The resulting shortages of PPE and ventilators depends on the demand and supply of PPE (A.9,A.11). Which supply strategies are applied and how many products are ordered, depends on the decision framework (A.13), which is based on the shortages of PPE and ventilators. Supply strategies are shown in Figures A.14, A.15, A.16, A.17, A.18, and A.19 The deliveries of PPE and ventilators feed back to the hospital capacities, as well as the shortage of PPE and ventilators.

Since the decision framework and supply strategies follow the same structure for PPE and ventilators, this appendix focuses on the case for ventilators.

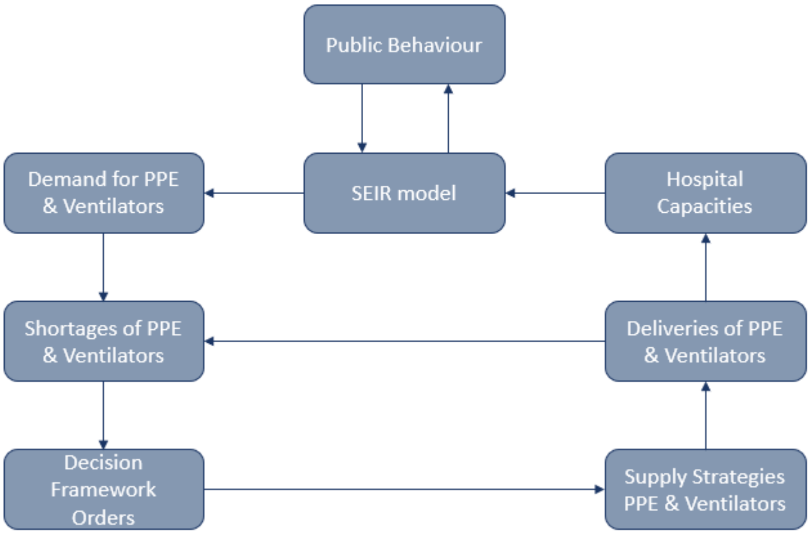

Figure A.1: Connections between subsystems modelled in Vensim

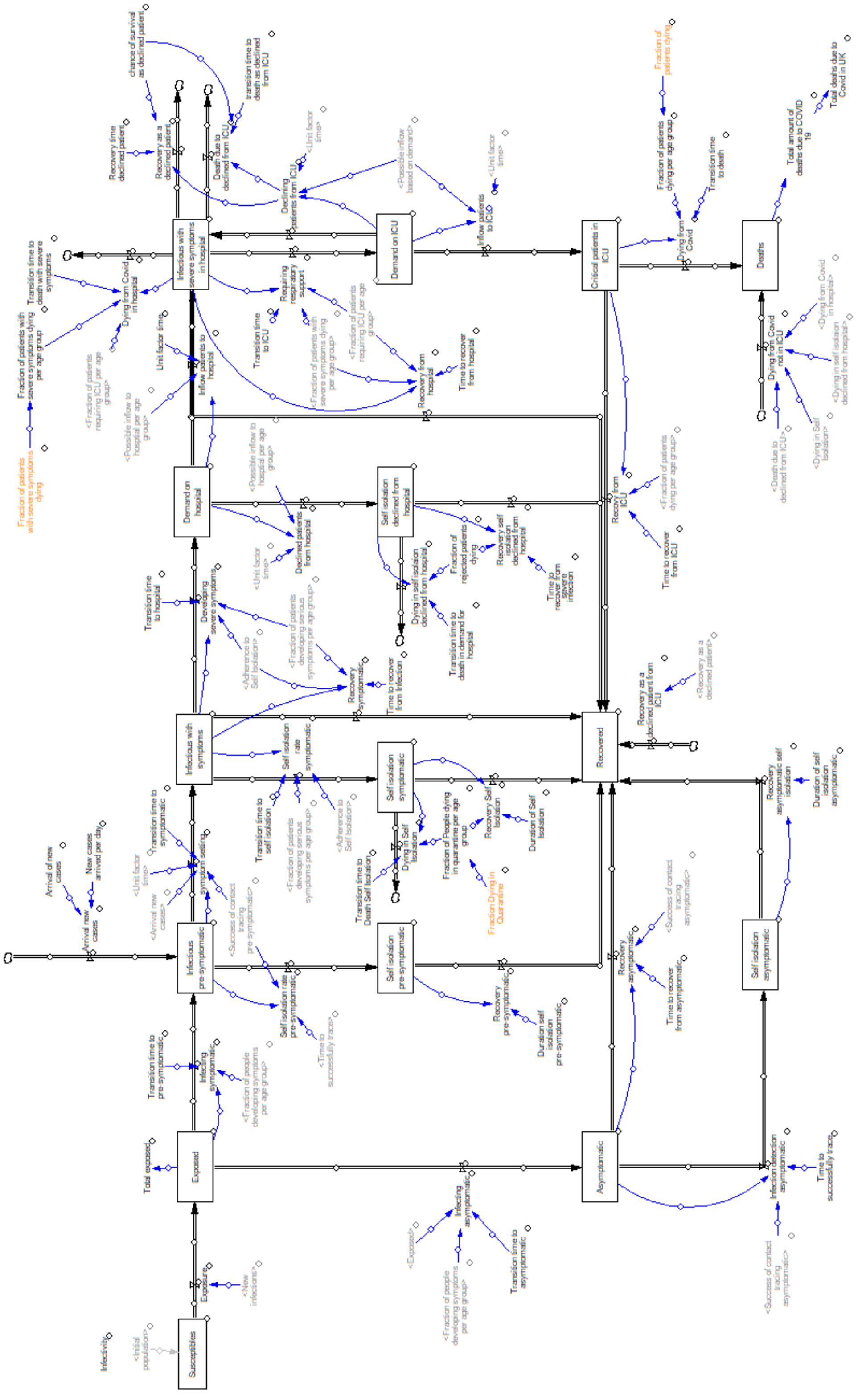

Figure A.2: SEIR model implemented in Vensim

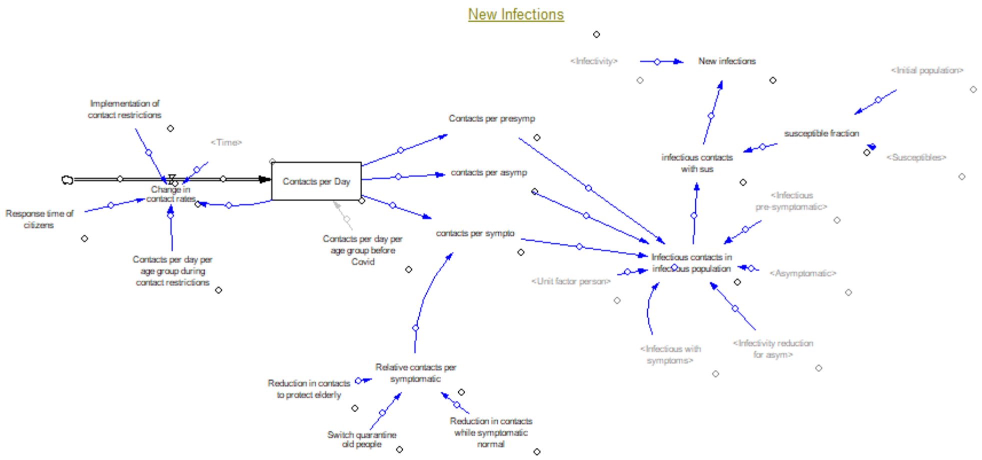

Figure A.3: Development of New Infections based on Contact Restrictions

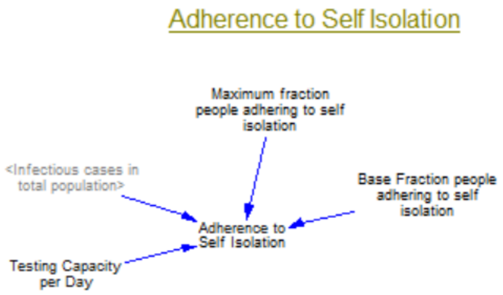

Figure A.4: Changes in Adherence to Self-Isolation

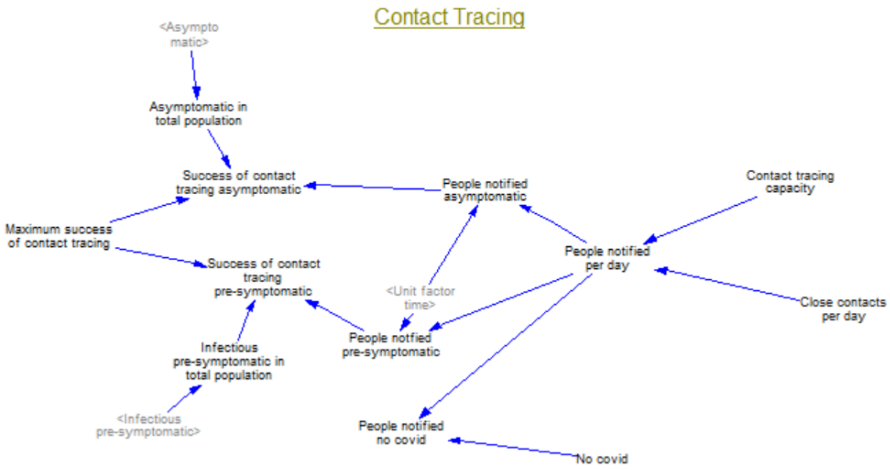

Figure A.5: Impact of Contact Tracing

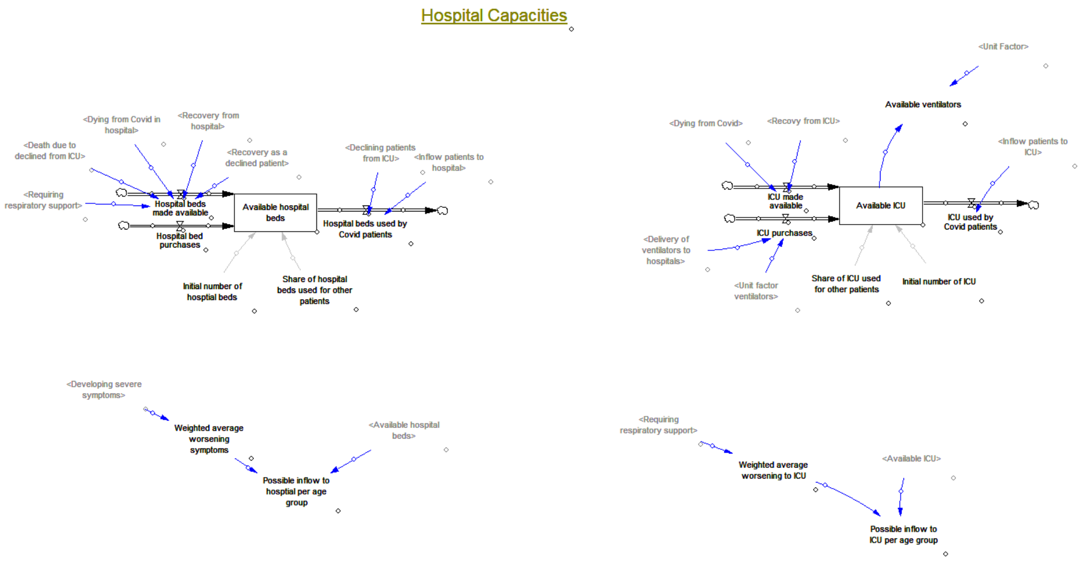

Figure A.6: Hospital Capacities

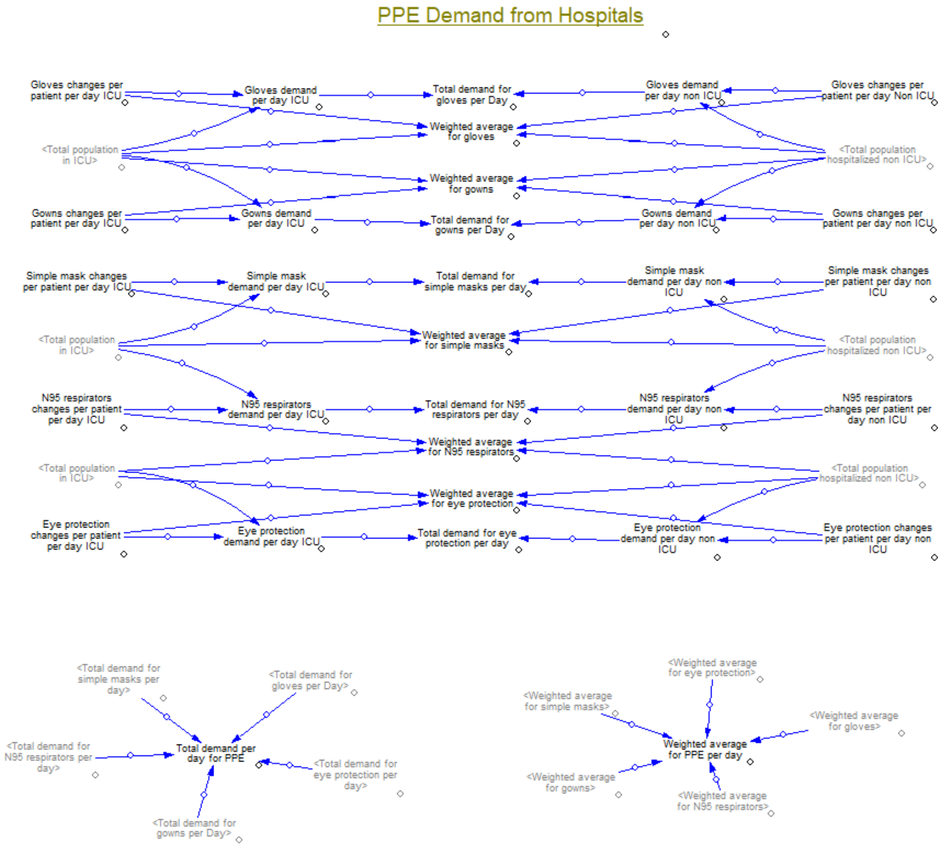

Figure A.7: Demand for PPE per Day

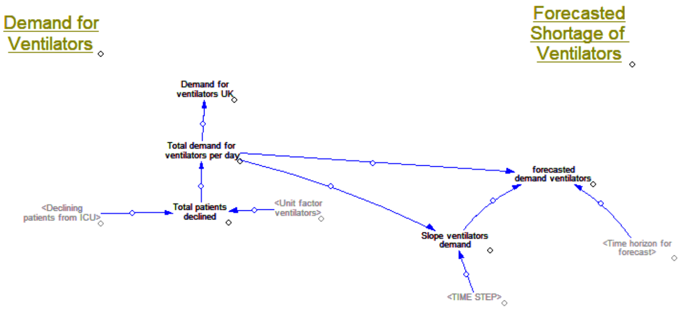

Figure A.8: Demand for Ventilators per day

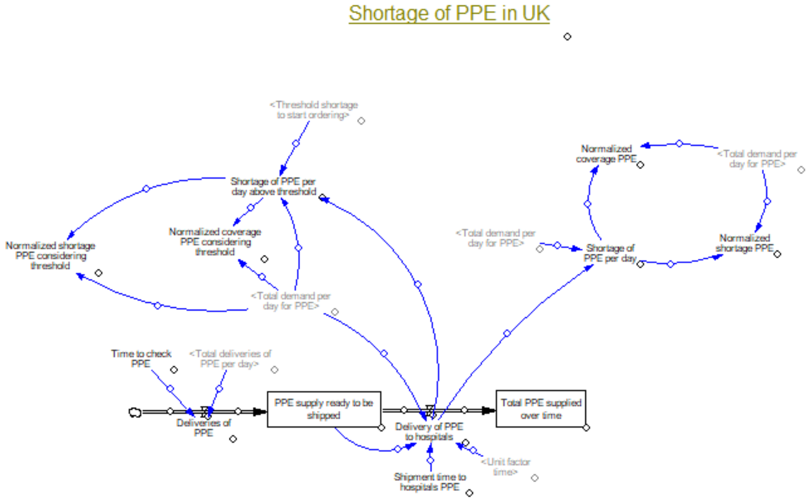

Figure A.9: Shortage of PPE per day based on the deliveries per day

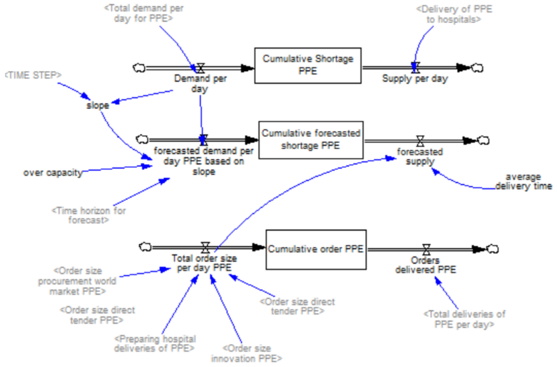

Figure A.10: Focus on forecasted demand of PPE.The entire figure gives an overview of cumulative orders.

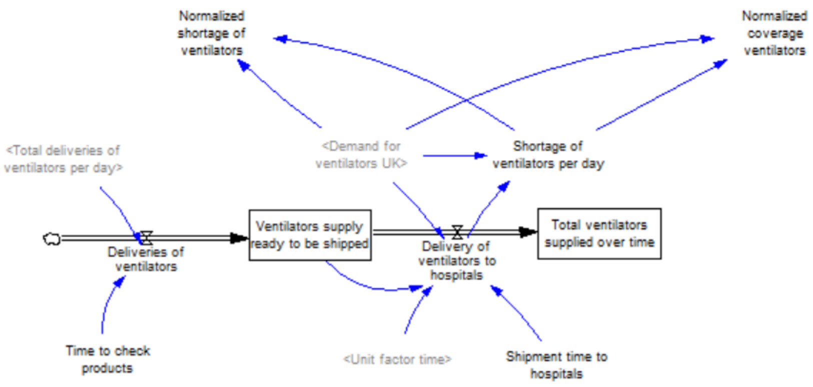

Figure A.11: Shortage of ventilators per day based on the deliveries per day

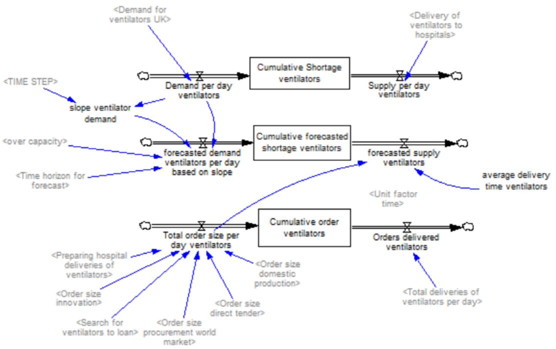

Figure A.12: Focus on forecasted demand for ventilators. The entire figure gives an overview of cumulative orders..

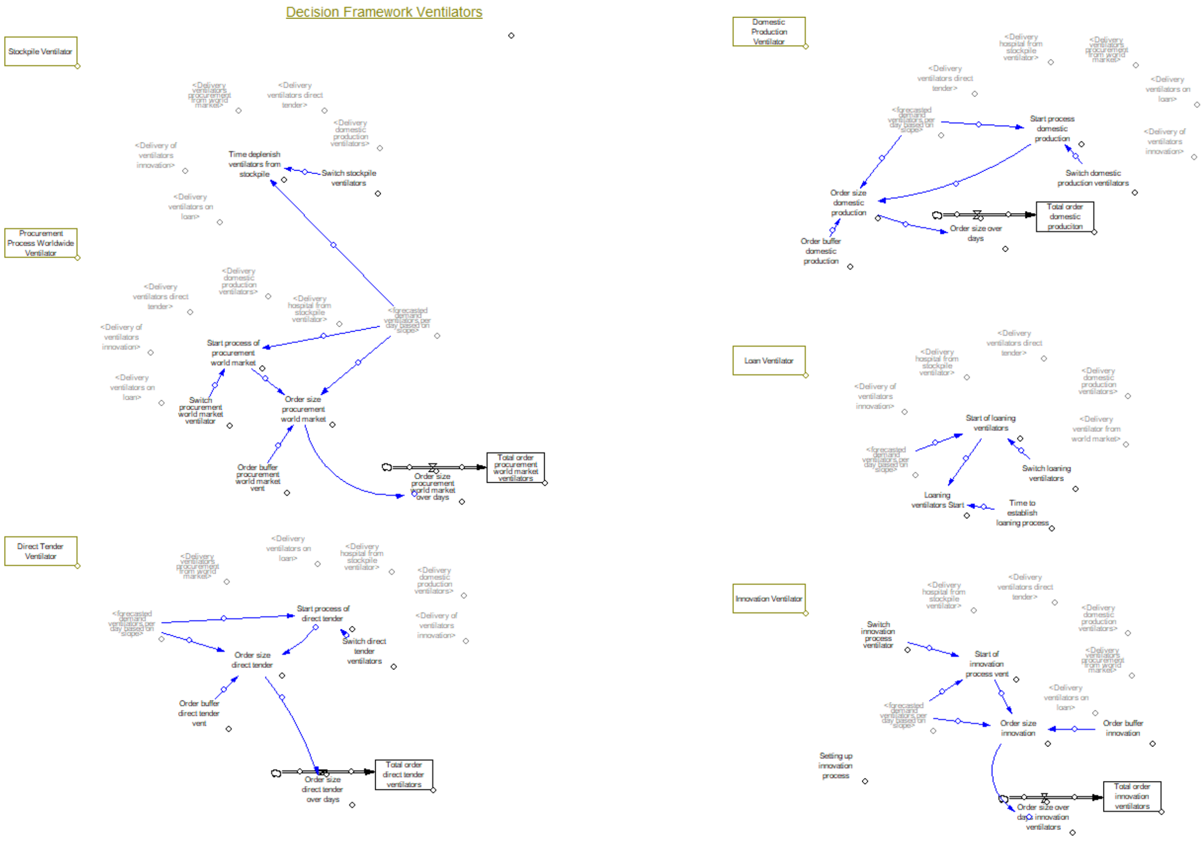

Figure A.13: Overview of decision framework for ventilators

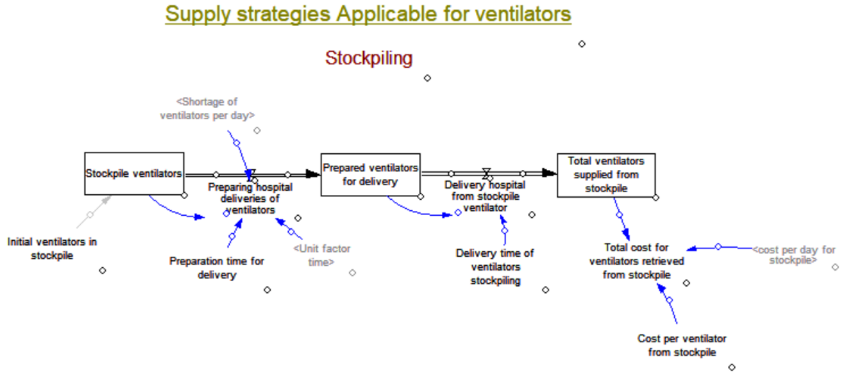

Figure A.14: Stockpiling as a supply strategy

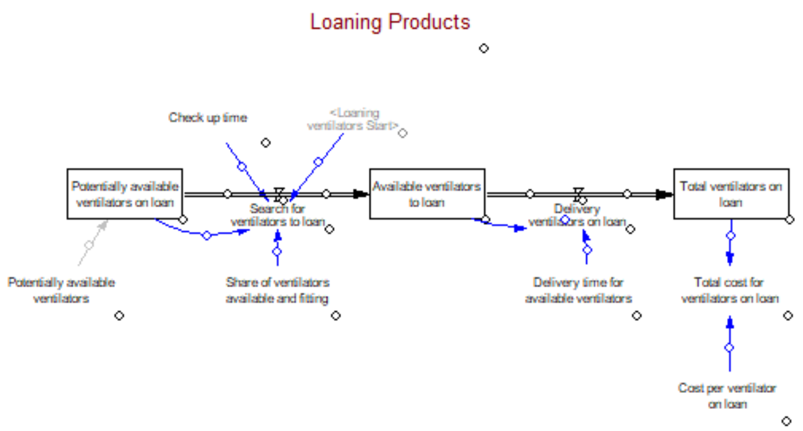

Figure A.15: Loaning ventilators as a supply strategy

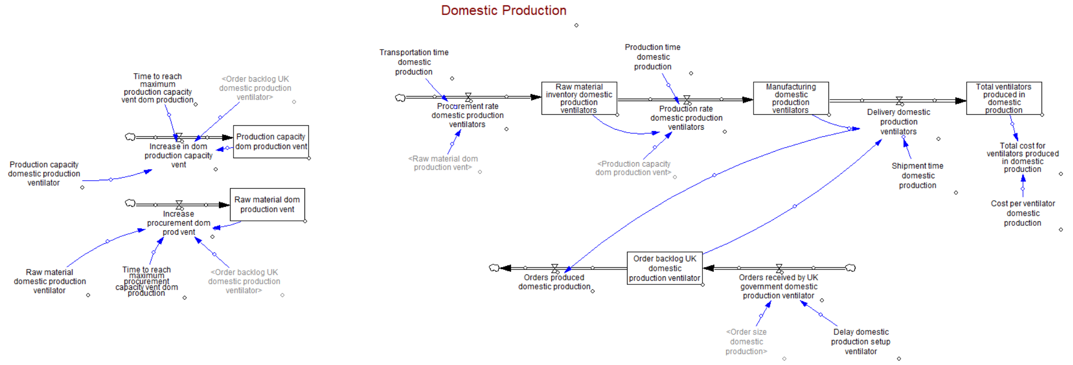

Figure A.16: Domestic production as a supply strategy

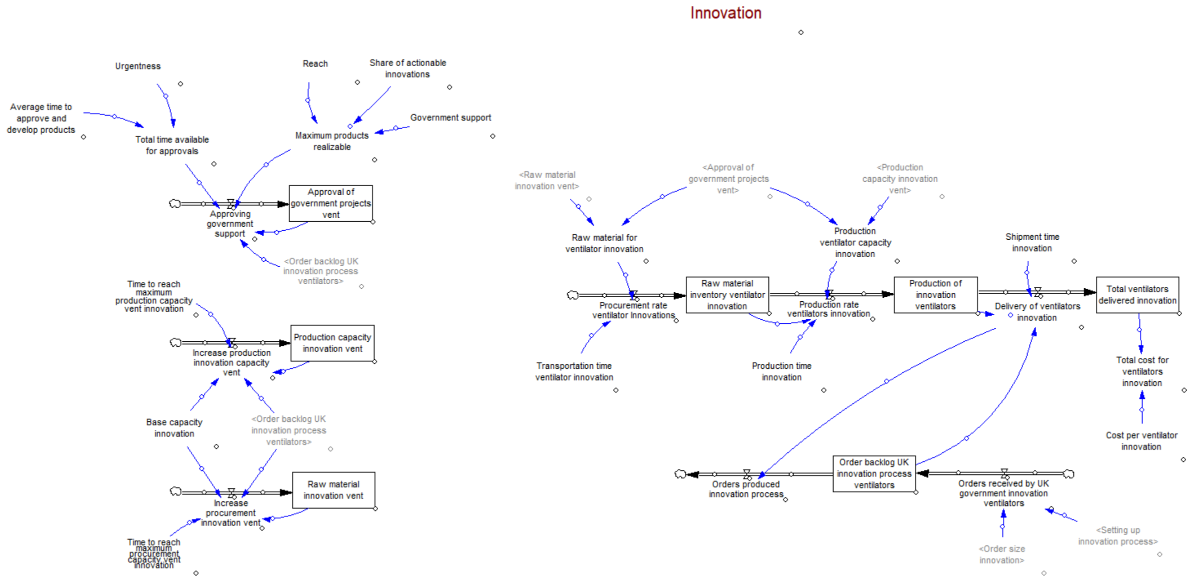

Figure A.17: Innovation as a supply strategy

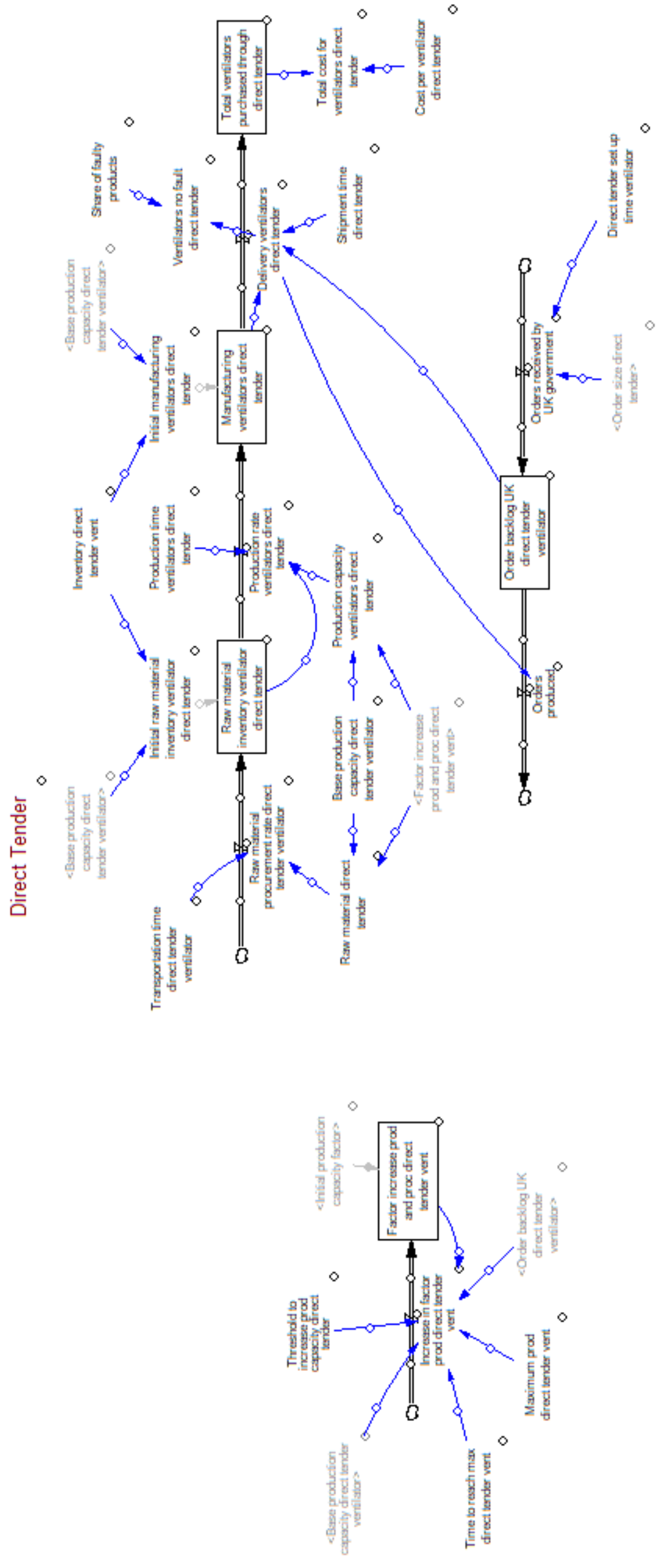

Figure A.18: Direct tender as a supply strategy

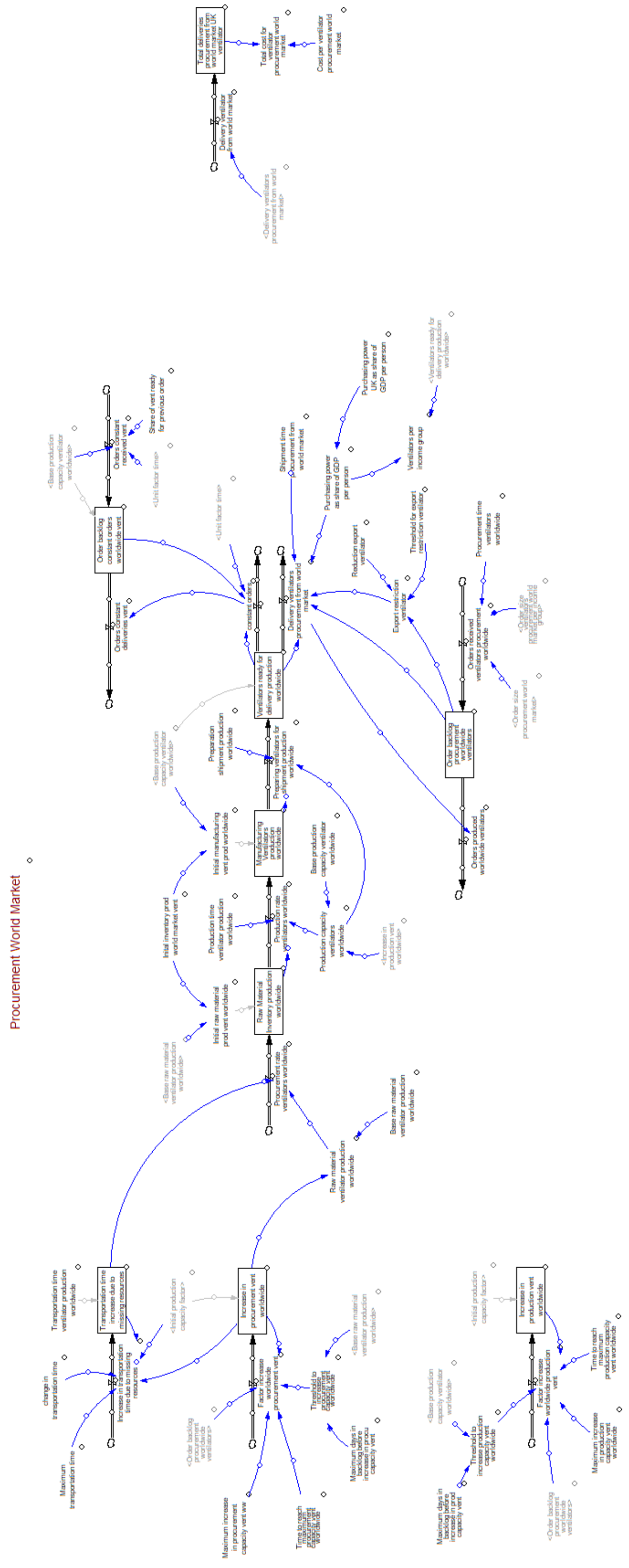

Figure A.19: Procurement from the world market as a supply strategy
