# Supplementary material for: Contributing to health system resilience during pandemics via purchasing and supply strategies: an exploratory system dynamics approach
Source: BMC Health Serv Res. 2024 Jan 24;24:130. doi: 10.1186/s12913-023-10487-7 (PMC10807148; doi:10.1186/s12913-023-10487-7)
Supplement: Supplementary file 2 — Additional file 2. [file 12913_2023_10487_MOESM2_ESM.pdf]

B

Outcomes, Uncertainties & Decision Levers in the System

The following appendix focuses on the outcomes of interest, decision levers and uncertainties within the system. The outcomes of interest are summarized as it is applicable. Decision levers and uncertainties for PPE and ventilators are presented separately.

The outcomes of interest are the same for ventilators and PPE. Hence, the outcomes of interest are summarized. In the directed search, only the total normalized coverage was considered. For the uncertainty analysis, all the listed outcomes were implemented to be able to provide a more thorough analysis if needed.

Table B.1: Outcomes of interest for PPE and ventilators

**Outcome**	**Maximize/ Minimize**	**Definition**
Total normalized coverage	Maximize	This variable tracks the total normalized coverage over time. This means the cumulative value is report. Ideally, the coverage equals 210, which would mean that at all days all patients can be served with ventilators, or all HCWs have sufficient PPE available to serve patients. During the analysis, only the value at the end of the simulation is of interest.
Shortage of PPE/ventilators per day		This variable reports the absolute shortage of medical equipment reported per day. This variable is of less interest, as it provides less insight to decision-makers, given they do not know the absolute demand for PPE or ventilators.
Normalized shortage of PPE/ventilators		This variable provides insight about how high the shortage per day is relative to the total demand. The normalized shortage can be insightful to decision-makers as it shows what percentage of patients or HCWs receive sufficient equipment per day. This variable highlights at what point of time the most shortages exist and when improvements start to be visible.

Normalized coverage PPE/ventilators		The normalized coverage of PPE and ventilators reports the coverage of PPE and ventilators in the same manner as the shortage of ventilators. This outcome is used as an input for the total normalized coverage.
Total cost for PPE/ventilators		This variable reports the costs of PPE or ventilators purchased and delivered considering all supply strategies. Furthermore, the maintenance costs for stockpiling medical equipment are considered.
Supply ready to be shipped		This outcome report the amount of supply that is ready to be distributed to hospitals in England per day. It shows decision-makers at what point of times buffers start existing.

The levers decision-makers have available are described in detail below. This means the decision lever the range of values for the decision lever is defined and the reasoning for the choice is presented. Where possible, sources are implemented. It is differentiated between the decision levers considered for PPE and ventilators. For PPE, only the decision levers relevant for simple masks and gloves are presented, as these two PPE products were used for the MORDM cycle.

Table B.2: Decision levers to affect the provision of PPE

**Decision lever**	**Range**	**Assumption Description**
Switch procurement world market PPE	(0, 1)	The switch determines whether a supply strategy is applied or not.It can be applied, if it takes the value 1. The value 0 indicates it is not applied.
Switch direct tender PPE	(0, 1)	
Switch domestic production PPE	(0, 1)	
Switch innovation PPE	(0, 1)	
Order buffer procurement world market PPE	(0.5, 3)	The order buffer indicates the overcapacity in ordering. It is assumed that decision-makers can order more products than needed. The UK for instance ordered as much as possible. A higher value presents a higher prioritization.
Order buffer direct tender PPE	(0.5, 3)	
Order buffer domestic production PPE	(0.5, 3)	
Order buffer innovation PPE	(0.5, 3)	
Delay domestic production PPE	(7, 60)	The delays regarding the set-up times for each process indicate when the first order is placed. The lowest times possible are 7 days as an equivalent to one week. Set-up times that take a shorter time initally, consider a lower minimum and a lower maximum value.
Direct tender set up time PPE	(7, 45)	
Set up time procurement PPE worldwide	(14, 50)	
Setting up innovation process PPE	(10, 45)	
Days in Stock	(7, 30)	The days in stock determine the threshold to start ordering PPE. The values considered are between a week and a month. No data could be found online regarding this value.
Number of patients	(10, 500)	The number of patients determine the threshold to start ordering PPE. The values considered are between a week and a month. No data could be found online regarding this value.
Time horizon for forecast	(5, 30)	Decision-makers can decide what time horizon is considered for shortages. A maximum range of one month is chosen. One week is considered a short-forecast horizon, 3 to 4 weeks can be considered a long horizon (Coroneo et al., 2022). Also, given the simplicity in calculating the forecast value no higher time horizon is considered.

Government budget for PPE	(0, 1)	The government can choose to a share of PPE innovation supply strategies. It is considered that the government support all proposed innovative projects to increase the attractiveness of innovative supply strategies.
Urgency	(0, 5)	Similarly, it is assumed that the government can influence how urgent action is taken to develop ideas regarding innovative PPE supply strategies. It is assumed that this value can range between 0 and 5. 5 is the maximum value considered on the scale.
Share of products expiring per day	(0, 0.0016667)	It was evident that expired products were stored in the stockpile for PPE (National Audit Office, 2020b). This value determines how high the share of PPE expiring each day is. A maximum value of 5% per month of the the entire inventory is assumed to be the maximum. No data was found about what share of the PPE stockpile expired in reality per month.
Share of stockpile available to hospitals	(0, 1)	This variable determines what share of PPE in the stockpile is delivered to hospital compared to other settings. In reality, 80% of the English stockpile went to hospitals (National Audit Office, 2020b).
Initial value for simple masks in stockpile UK	(0, 468000000)	3 times the advised value mentioned in reports by National Audit Office (2020b) were considered to be the maximum PPE to be stored. This value may be difficult to reach in reality due to costs.
Initial value for gloves in stockpile UK	(0, 1079700000)	
Preparation time for delivery PPE	(1, 10)	The values refer to the delay in days in the operation of the stockpile, as it was the case (National Audit Office, 2020b).
Delivery time of PPE stockpiling	(1, 21)	
Time to check PPE	(1, 5)	The values refer to delay in days in checking products and their distribution.
Shipment time to hospitals PPE	(1, 10)	

Table B.3: Decision levers for the provision of ventilators

**Decision lever**	**Range**	**Assumption Description**
Switch procurement world market ventilator	(0, 1)	The switch determines whether a supply strategy is applied or not. It can be applied, if it takes the value 1. The value 0 indicates it is not applied. Stockpiling is assumed to be applied.
Switch direct tender ventilators	(0, 1)	
Switch innovation process ventilator	(0, 1)	
Switch loaning ventilators	(0, 1)	
Switch domestic production ventilators	(0, 1)	
Direct tender set up time ventilator	(7, 45)	The delays regarding the set-up times for each process indicate when the first order is placed. The lowest times possible are 7 days as an equivalent to one week. Set-up times that take a shorter time initally, consider a lower minimum and a lower maximum value. More complicated procurement processes come with a higher maximum value.
Check up time	(5, 21)	
Delay domestic production setup ventilator	(7, 60)	
Procurement time ventilators worldwide	(15, 60)	
Time to establish loaning process	(3.5 , 21)	

Order buffer procurement world market vent	(0.5, 3)	The order buffer indicates the overcapacity in ordering. It is assumed that decision-makers can order more products than needed. The UK for instance ordered as much as possible. A higher value presents a higher prioritization.
Order buffer direct tender vent	(0.5, 3)	
Order buffer domestic production	(0.5, 3)	
Order buffer innovation	(0.5, 3)	
Initial ventilators in stockpile	(0, 10000)	No data was available regarding the number of ventilators stored. The range is based on the maximum demand plus an assumed bugger.
Delivery time of ventilators stockpiling	(1, 14)	The values refer to the delay in days in the operation of the stockpile (National Audit Office, 2020b). A delay is also possible for the delivery.
Preparation time for delivery	(1, 10)	
Time horizon for forecast	(5, 30)	One week is considered a short-forecast horizon, 3 to 4 weeks can be considered a long horizon (Coroneo et al., 2022).Given the simplicity in calculating the forecast value no higher time horizon is considered.
Urgency	(0, 5)	It is assumed that the government can influence how urgent action is taken to develop ideas regarding innovative ventilator supply strategies. It is assumed that this value can range between 0 and 5. 5 is the maximum value considered on the scale.
Government support	(0, 0.1)	The government can choose to a share of ventilator innovation supply strategies. It is considered that the government support cannot support all proposed projects, as the development of ventilators is often more expensive. The number refers to the amount of ventilator designs approved during the ventilator challenge versus its reach (National Audit Office, 2020a).
Time to check products	(1, 5)	The values refer to delay in days in checking products and their distribution.
Shipment time to hospitals	(1, 10)	

The uncertainties considered for the directed search of PPE (simple masks and gloves) and ventilators are presented in Table B.4 and B.5. Next to the range considered for MORDM, this table also includes the assumed value in the original Vensim model, as well as the source or assumption that is supporting the assumed value and the range of values considered.

Table B.4: Uncertainties considered for PPE

Uncertainty	Assumed Value	Range	Source/Reason
Gloves changes per patient per day ICU	170	(85,250)	The assumed value is based on the assumptions provided by Johns Hopkins Center for Health Security (2020). For the uncertainty analysis, a value of from 50% to 150% of the orignal assumptions to consider for changes.
Gloves changes per patient per day Non ICU	80	(40,120)	
Gowns changes per patient per day ICU	20	(10, 30)	
Gowns changes per patient per day non ICU	20	(10, 30)	
Simple mask changes per patient per day ICU	10	(5, 15)	
Simple mask changes per patient per day non ICU	10	(5, 15)	

N95 respirators changes per patient per day ICU	3	(2, 6)	
N95 respirators changes per patient per day non ICU	2.6	(1.3 , 3.9)	
Eye protection changes per patient per day ICU	6	(3, 9)	
Eye protection changes per patient per day non ICU	6	(1.3 , 3.9)	
Infectivity	0.08	(0.02, 0.2)	Based on the assumption, that the infectivity of a coronavirus varies, as it did among different wave of COVID-19.
Transportation time domestic production PPE	7	(1, 14)	Refers to the delay in material procurement. It is assumed that the delivery of products can be up to 14 days late due to raw material shortages.
Base raw material procurement eye protection domestic production	12000	(20000, 240000)	No information was provided regarding production values. Hence high varieties were chosen. Products that are easier to produce have higher ranges (gloves, simple masks). Furthermore, orders by the English health system were considered as corner points (National Audit Office, 2020b), as well as production capacities of other countries.
Base raw material procurement simple masks domestic production	598800	(20000, 900000)	
Base raw material procurement N95 respirators domestic production	17400	(5000, 50000)	
Base raw material procurement gowns domestic production	120000	(20000, 240000)	
Base raw material procurement gloves domestic production	698630	(144000, 1440000)	
Shipment time domestic production PPE	5	(1,21)	Department of Health and Social Care (2020) assures deliveries within 5 days for PPE from stockpile. The assumption is made that the same delivery time is considered for domestic supply strategies. Additional delays are considered as an uncertainty.
Production time domestic production PPE	1.5	(1,10)	Refers to appearing delays in the production, likely to happen due to learning process in production.
Time to reach maximum production capacity PPE dom production	45	(5, 90)	Refers to the time it takes to reach the maximum production or procurement capacity. Assumption, that it can take up to three months to reach maximum. (Based on shipment times)
Time to reach maximum procurement capacity PPE dom production	30	(5, 90)	
Transportation time direct tender PPE	5	(1,21)	Delays in purchasing raw material for production of PPE. Such delays can increase due to raw material shortages (OECD, 2020).
Threshold to start direct tender PPE	7	(1,21)	This value determines when suppliers reached through direct tender start to increase their production. It refers to the existing order backlog.
Time to reach max direct tender	21	(1,90)	Time it takes to reaches maximum increase in production & procurement capacity

Maximum prod direct tender PPE	4	(1,12)	Based on the increase possible for Chinese producers (OECD, 2020).
Base raw material eye protection capacity direct tender	199600	(20000, 400000)	Assumed to be 5% of production & procurement capacity worldwide. Assumption that Enlgish health system can reach up to 10% of capacity purchasing products from world market.
Base raw material simple masks capacity direct tender	998000	(50000, 2000000)	
Base raw material N95 respirators capacity direct tender	29000	(10000, 100000)	
Base raw material gowns capacity direct tender	199600	(20000, 400000)	
Base raw material gloves capacity direct tender	3.49315 e+07	(800000, 50000000)	
Shipment time direct tender PPE	45	(14, 120)	It is assumed products are flown into the country, as the delivery is guaranteed and the price per piece is higher. This takes between 1 and 3 months (Gandrup-Marino et al., 2021).
Share of faulty PPE	0.15	(0, 0.5)	7& of ordered PPE was unusable in the first year of Covid-19 (Public Accounts Committee - House of Commons, 2021). Assumed to reach up to 50%.
Reach PPE	100	(0, 300)	Maximum businesses that can be reached. No information was available. Hence, the values is assumed to be uncertain. Values above 300 return unrealistic production capacities.
Production time Innovation PPE	2	(1, 7)	Delays in production of suppliers using innovative supply strategies. Uncertainty range assumed, as no data was found.
Transportation time PPE innovation	7	(3.5, 21)	Delays in prcorument of raw material. Likely to occur as suppliers often procure smaller amounts of raw material, and suppliers are new to production. Hence, higher uncertainty compared to other delays in procurement.
Shipment time innovation PPE	5	(3.5, 14)	Department of Health and Social Care (2020) assures deliveries within 5 days for PPE from stockpile. The assumption is made that the same delivery time is considered for domestic supply strategies. Fewer than domestic production because of smaller the design of innovative supply strategies.
Base raw material eye protection capacity innovation PPE	1000	(500, 30000)	Refers to the production capacity per idea. Uncertainty ranges based on information found in articles, plus additional range where applicable (Cates, 2020; Dixon, 2020; Pagliacolo & Pavka, 2020).
Base raw material simple masks capacity innovation PPE	7200	(3400, 34000)	
Base raw material N95 respirators capacity innovation PPE	1000	(3400, 25200)	
Base raw material gowns capacity innovation PPE	2000	(500, 30000)	
Base raw material gloves capacity innovation PPE	20000	(5000, 160000)	

Average time to approve and develop PPE	60	(15, 120)	Assumption, based on time it took to develop ventilators (National Audit Office, 2020a). Range can be smaller or higher depending on PPE and innovation.
Time to reach maximum production capacity PPE innovation	30	(5, 90)	Time it takes to reach their maximum capacity. The uncertainty range considers the length of the pandemic.
Time to reach maximum procurement capacity PPE innovation	30	(5, 90)	
Base raw material procurement eye protection worldwide	3.992e+06	(1000000, 8000000)	Assumed values consider the available data where possible (Bhutta & Santhakumar, 2016; OECD, 2020; Statista, 2022). Uncertainty ranges assume up to 3 times the current output.
Base raw material procurement simple masks worldwide	1.996e+07	(10000000, 60000000)	
Base raw material procurement N95 respirators worldwide	580000	(120000, 1200000)	
Base raw material procurement gowns worldwide	3.992e +06	(1000000, 8000000)	
Base raw material procurement gloves worldwide	6.9863e +08	(200000000, 1400000000)	
Preparation shipment PPE production worldwide	1	(1, 10)	Delay in preparing shipments for delivery. Interruptions of production are more likely due to health-related crisis.
Threshold for export restriction PPE		(1000000, 100000000)	Value determines at what order backlog of orders due to Covid-19, the export is restricted. As no information was available, a wide range was considered.
Delayed shipment time	90	(30, 360)	Lead times of up to 9 months were possible (Gandrup-Marino et al., 2021).
Normal shipment time	21	(7, 45)	Delivery times from PPE producers in China ranges between 15 to 30 days (HisoMedical, 2020). A wider range was considered to account for suppliers located in other countries as well.
Reduction export PPE	1	(0, 1)	Value determines to what percentage the export is reduced. It can take on any value between 0 and 1.
Maximum increase in procurement capacity PPE	10	(1, 20)	Assumption based on production icnrease by Chinese suppliers (OECD, 2020). Considered uncertainty range based on length of simulation run.
Time to reach maximum procurement capacity PPE worldwide	120	(14, 210)	
Maximum days in backlog before increase in procurement capacity	10	(1, 45)	
Maximum increase in production capacity PPE	10	(1, 20)	Assumption based on production icnrease by Chinese suppliers (OECD, 2020). Considered uncertainty range based on length of simulation run.
Time to reach maximum production capacity PPE worldwide	120	(14, 210)	
Maximum days in backlog before increase in prod capacity	14	(1, 20)	Determines when suppliers increase their production. No knowledge available.
Share of PPE ready for previous order	0.6	(0.2, 1)	Assumption, that share of products goes to orders that were placed before Covid-19 hit the system.

Maximum transportation time PPE procurement world market	8	(1, 20)	Assumption based on production icnrease by Chinese suppliers (OECD, 2020). Considered uncertainty range based on length of simulation run.
change in transportation time PPE	21	(7, 60)	

Table B.5: Uncertainties considered for ventilators

**Uncertainty**	**Assumed Value**	**Range**	**Source/Reason**
Production time domestic production	1	(1,5)	Delay in domestic production.
Transportation time domestic production	1.5	(1,10)	Delay in procurement of materials for domestic production. Higher than the delay in domestic production, as it may be more difficult to reach raw suppliers ad hoc.
Shipment time domestic production	3	(1,21)	Department of Health and Social Care (2020) assures deliveries within 5 days for PPE from stockpile. The assumption is made that the same delivery time is considered for domestic supply strategies. Additional delays are considered as an uncertainty. Higher for ventilators, since transportation may be more difficult.
Time to reach maximum production capacity vent dom production	14	(5,30)	Assumption based on information about ventilator challenge provided by Leggett (2020). Assumption is that it also includes raw materials.
Time to reach maximum procurement capacity vent dom production	10	(5,30)	
Production capacity domestic production ventilator	214	(50,430)	
Raw material domestic production ventilator	214	(50,430)	
Delivery time for available ventilators	5	(1,21)	Department of Health and Social Care (2020) assures deliveries within 5 days for PPE from stockpile. The assumption is made that the same delivery time is considered for domestic supply strategies. Additional delays are considered as an uncertainty. Higher for ventilators, since transportation may be more difficult.
Share of ventilators available and fitting	0.7	(0,1)	Refers to share of ventilators suitable to be loaned, it can take any value between 0 and 1
Potentially available ventilators	1700	(0,2400)	Refers to the ventilators that may be suitable to loan. Assumption based on National Audit Office (2020a)
Transportation time direct tender ventilator	1.5	(1,10)	Delays may occur in the procurement of raw material for production of ventilators.
Production time ventilators direct tender	1	(1,10)	Delays may occur due Covid-19 in the production.

Base production capacity direct tender ventilator	250	(50,500)	Assumed to be similar to the values of domestic production, as it is difficult to order products.
Maximum prod direct tender vent	5	(1,10)	Assumption
Shipment time direct tender	30	(14,120)	Range based on available information about shipment times of PPE.
Share of faulty products	0.05	(0, 0.5)	Assumption that up to half of purchased ventilators can be useless, based on reports about unusable ventilators (Campbell, 2020).
Reach	2000	(0,2000)	Based on the number of participating businesses (National Audit Office, 2020a).
Production time innovation	1	(1,7)	Assumption that production may be delayed.
Share of actionable innovations	0.1	(0.01, 0.2)	Share of actionable innovations is assumed to lower for ventilators, as they are more complicated to produce.
Transportation time ventilator innovation	1.5	(1,10)	Assumption that delays may exist in the delivery of raw material for innovative suppliers.
Shipment time innovation	5	(1,21)	Department of Health and Social Care (2020) assures deliveries within 5 days for PPE from stockpile. The assumption is made that the same delivery time is considered for domestic supply strategies. Additional delays are considered as an uncertainty. Higher for ventilators, since transportation may be more difficult.
Average time to approve and develop products	45	(15,120)	Higher range of values compared to PPE chosen because it is more complicated to produce. Range is based on information from ventilator challenge (National Audit Office, 2020a).
Time to reach maximum production capacity vent innovation	30	(10,90)	No information available regarding both variables. Range of uncertainty is choasen based on length of pandemic
Time to reach maximum procurement capacity vent innovation	21	(10,90)	
Base capacity innovation	10	(1,30)	Assumed to be very low, as single project may only produce a few ventilators per week.
Production time ventilator production worldwide	1	(1,10)	Delays may occur due to health-related crisis.
Base raw material ventilator production worldwide	850	(430,1720)	Assumption, that US produces 1/3 of ventilator supply, and based on the information about ventilator production in US (Statista, 2020).
Preparation shipment production worldwide	1	(1,10)	Delays may occur due to health-related crisis.
Purchasing power UK as share of GDP per person	0.0881	(0.05, 0.33)	Refers to the purchasing power of England. Based on the GDP of the UK, compared to other countries.

Shipment time procurement from world market	30	(14,90)	Shipment times may be delayed (similar assumption as with PPE)
Maximum increase in procurement capacity vent ww	10	(1,15)	Based on information about ventilator production in US (Statista, 2020).
Time to reach maximum procurement capacity vent worldwide	210	(60,480)	Based on information about ventilator production in US (Statista, 2020).
Maximum increase in production capacity vent worldwide	10	(1,15)	Based on the data derived from (Statista, 2020).
Reduction export ventilator	1	(0,1)	Export restrictions may apply to ventilators.
Time to reach maximum production capacity vent worldwide	240	(60,480)	Due to the complexity of the ventilator production, it takes more time to reach full production capacity.
Maximum days in backlog before increase in prod capacity vent	30	(14,60)	No information available regarding both variables.
Maximum days in backlog before increase in procu capacity vent	21	(14,60)	
Share of vent ready for previous order	0.7	(0.2,1)	Assumption that share of ventilators is delivered to customers whose demand did not result from COVID-19.
Maximum transportation time	3	(1,3)	No information available regarding both variables.
change in transportation time	21	(7,60)	
